# Simple non-fused electron acceptors for efficient and stable organic solar cells

**DOI:** 10.1038/s41467-019-10098-z

**Published:** 2019-05-14

**Authors:** Zhi-Peng Yu, Zhi-Xi Liu, Fang-Xiao Chen, Ran Qin, Tsz-Ki Lau, Jing-Lin Yin, Xueqian Kong, Xinhui Lu, Minmin Shi, Chang-Zhi Li, Hongzheng Chen

**Affiliations:** 1 State Key Laboratory of Silicon Materials, MOE Key Laboratory of Macromolecular Synthesis and Functionalization, Department of Polymer Science and Engineering, Zhejiang University, 310027 Hangzhou, P. R. China; 2 Department of Physics, The Chinese University of Hong Kong, New Territories, 999077 Hong Kong P. R. China; 3 Department of Chemistry, Zhejiang University, 310027 Hangzhou, P. R. China

**Keywords:** Materials science, Materials for energy and catalysis, Solar cells

## Abstract

The flexibility in structural design of organic semiconductors endows organic solar cells (OSCs) not only great function-tunabilities, but also high potential toward practical application. In this work, simple non-fused-ring electron acceptors are developed through two-step synthesis from single aromatic units for constructing efficient OSCs. With the assistance of non-covalent interactions, these rotatable non-fused acceptors (in solution) allow transiting into planar and stackable conformation in condensed solid, promoting acceptors not only feasible solution-processability, but also excellent film characteristics. As results, decent power conversion efficiencies of 10.27% and 13.97% can be achieved in single and tandem OSCs consisting of simple solution-cast blends, in which the fully unfused acceptors exhibit exceptionally low synthetic complexity index. In addition, the unfused acceptor and its based OSCs exhibit promising stabilities under continuous illumination. Overall, this work reveals valuable insights on the structural design of simple and effective electron acceptors with great practical perspectives.

## Abstract (web summary)

Non-fullerene electron acceptors have pushed the efficiency of organic solar cells up to 15% but they all contain fused rings and are inconvenient to synthetic access. Here Yu et al. develop fully unfused acceptors featuring non-covalent intramolecular interactions, high efficiencies and high stability.

## Introduction

As one of the promising clean energy solutions, the organic solar cell (OSC) possesses unique features distinct from inorganic ones, such as lightweight and flexibility, as well as the great function tunabilities rooting from the structural versatilities of organic semiconductors^1,2^. During the past few decades, OSC has experienced continuous advancement with the fast evolution of electroactive molecules. Once, OSCs commonly employed the bulk heterojunction (BHJ) of p-type polymers and/or molecules with n-type fullerene electron acceptors^3–6^. Recent breakthrough of OSC performance was made possible^1,2,7–9^, with the dawn of fused-ring electron acceptor (FREA) at 2015^10,11^, wherein FREA typically adapts acceptor–donor–acceptor (A–D–A) molecular architecture with a fused ladder-type core (D)^2^. Fused ladder structures refer to those chemical-bonds-reinforced multiple membered-ring structures (usually more than five rings), which allow potentially reducing the reorganization energy of molecules^12,13^. The recent development of highly efficient FREAs, all consisting of fused ladder cores, such as IDTBR^14^, IDIC^15^, ITIC^10^, IHIC^16^, IEICO^12^, ATT-2^17^, BT-CIC^18,19^, DTPC-IC^20^, CO*i*8DFIC^21^, etc.^2,8,22^, enables BHJ layers to effectively utilize solar photons from visible to near-infrared range (NIR). Power conversion efficiencies (PCEs) of the OSCs with FREAs have nowadays reached over 14%^8^ for single junction and 17%^23^ for tandem OSCs, respectively. Although exciting progresses have been made, the derivation of FREAs has surprisingly expanded into rather complex structures, ranging from five to seven, nine, and eleven- (or more) membered rings.

While the level of OSC performance is revalent to the practical application, significant challenges, such as the accessibility of effective materials and devices, should be put under consideration for research activities, which is closely related to the scalability and costs of OSCs^14,24,25^. So far, the access of multi-ring-fused ladder structure inevitably involves synthetic complexities. A typical example is the indacenodithiophene (IDT) unit, the simplest fused-ring electron-donating core for FREAs reported so far, which is typically synthesized over 6 (or 4) steps, depending on either aliphatic (or aromatic) side chains attached on the bridge *sp*^3^-carbon up-pointing and down-pointing fashion. The synthetic complexities of the molecule are inversely proportional to the industrial figure of merit for OSCs^24,26^, since multi-step synthesis and purification rapidly increase the material costs. These issues would potentially undermine the practical perspectives of OSCs. Therefore, despite a large family of electron acceptors developed, there is still a strong need to explore the new structural design of simple and effective molecules, for accessing efficient and low-cost OSCs.

Bearing this in mind, electron acceptors with partially^17,27–29^ or fully unfused backbones^30^, have recently been explored, wherein the noncovalent intramolecular interactions^29,31–33^ are employed to mediate the planarity of these molecular structures. If performance insufficiency is overcome, efficient OSCs with easy accessibility of key active components could be expected. Therefore, it is highly interesting to explore the design strategy and structure–property relation of non-fused-ring acceptors (NFRAs), particularly to embed excellent optoelectronic and stacking properties for the unfused (and potentially rotatable) molecules, which allows improving their BHJ characteristics and photovoltaic performance.

Herein, three exceptionally simple electron acceptors, PTICH, PTIC, and PTICO NFRAs, consisting of unfused 2,2′-(2,5-dialkyloxy-1,4-phenylene) dithiophene (PT) core and fluorinated 1,1-dicyanomethylene-3-indanone (DFIC) terminals, are developed through two-step synthesis without column purification from single aromatic units for constructing efficient OSCs. Through theoretical and experimental investigations, we reveal that NFRAs with rotatable conformation in solution could be restrained into planar and stackable conformation in condensed solid. The reasons are due to the presence of intramolecular O–H interaction among the PT core and steric hindrance between substituted thiophene and terminal DFIC (3-position substituent on thiophene: hexyl of PTIC and hexyloxy of PTICO, rather than H of PTICH), wherein the former reinforces backbone planarity, and the latter prevents the structural isomerization of the terminal double bond on NFRAs. Meanwhile, as evidenced from solid-state nuclear magnetic resonance (SSNMR) and grazing-incidence wide-angle X-ray scattering (GIWAXS), dense end-to-end stacking of NFRAs is observed with closer π–π distance than that of FREA, ID4F. These promote NFRAs with the improved optoelectronic properties in solids.

Among OSCs containing BHJs made of NFRAs and PBDB-TF polymer, decent PCEs of 10.27% and 13.97% can be achieved for the single and tandem OSCs consisting of PTIC-based BHJs, in which PTIC exhibits exceptionally low synthetic complexity index. In addition, NFRA itself, i.e., PTIC and NFRA-based OSCs exhibit promising stabilities under continuous 1-sun-equivalent illumination. Overall, we demonstrate that exceptionally simple acceptors with desirable optoelectronic properties in solids can lead to efficient and stable OSCs. This study reveals valuable insights on the structural design of simple and effective electron acceptors with great practical perspectives.

## Results

### Synthesis of NFRAs and molecular monformation

**Fig. 1:**
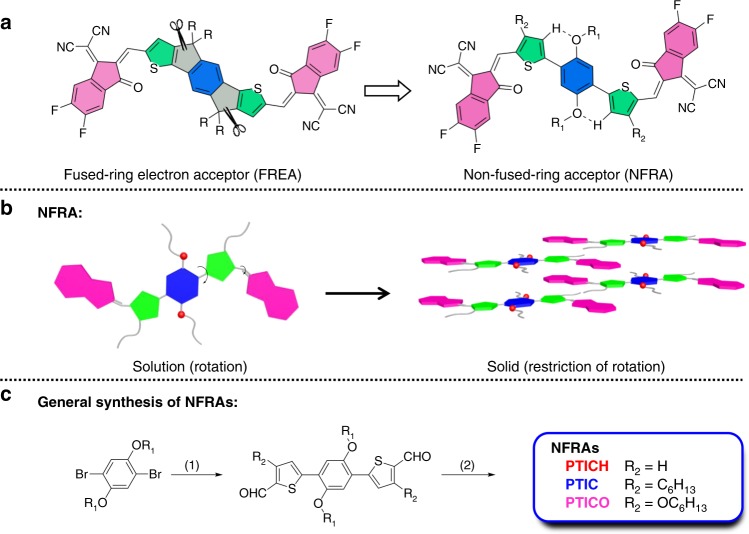
Non-fused-ring acceptors (NFRAs) and their synthetic route. **a** The chemical structure of FREA and NFRA. **b** The schematic presentation of NFRA conformation rearrangement from solution to solid state. **c** Two-step synthetic route for NFRAs. Here R_1_ is 2-hexyldecyl. Reacted condition: (1) 3-substituent-2-thenaldehyde, palladium-catalyzed C–H activation coupling and (2) DFIC, Knoevenagel condensation

The fully non-fused acceptors, PTICH, PTIC, and PTICO, are designed and achieved with a two-step synthetic route (Fig. 1), which are generally more convenient than the synthesis of FREAs (Supplementary Figs. 1 and 2). The chemical structures of the studied NFRAs and polymer donors, along with a fused-ID4F acceptor are shown in Supplementary Figs. 3 and4. PT-CHOs were first constructed by Palladium (II)-catalyzed C–H activation coupling reactions between 1,4-dibromo-2,5-bis((2-hexyldecyl)oxy) benzene and thiophene-2-carbaldehydes, featured with atomic economy. Target products were finally accessed from Knoevenagel condensation of PT-CHOs and DFIC. It is worthy to note that NFRAs were purified by recrystallization without the need of silica gel column, featured with the simple and scalable work-up processes. The final products were characterized by ^1^H-NMR, ^13^C-NMR, and mass spectra. The synthetic complex (SC) index of these NFRAs was also calculated according to reported methodologies^24,34^, wherein the SC index of PTIC is around 55%, much smaller than that of ID4F (97%) and other compared FREAs (generally beyond 80%) (Supplementary Table 1). Essentially, the smaller SCs for NFRAs were attributed from intrinsically short synthetic routes and ease of purification.

**Fig. 2:**
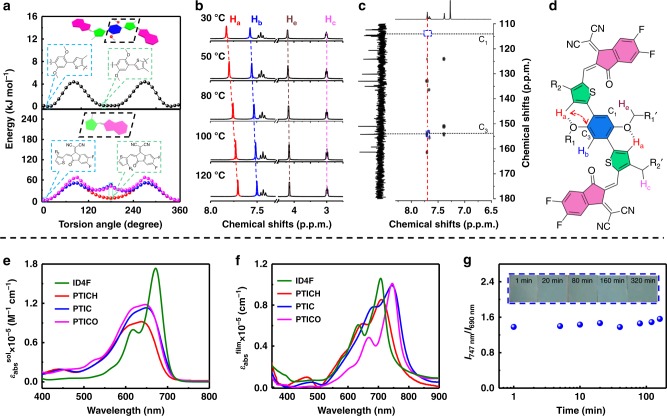
Molecular conformation and optical properties. **a** Possible rotamers and their energy–torsion angle (*E*–*θ*) curves: black curve for PT rotamer (left structure for O–H geometry and O–S geometry at the right); TICH rotamer (red), TIC rotamer (blue), and TICO rotamer (pink). **b** Temperature-dependent NMR of PTIC in d_4_-C_6_D_4_Cl_2_. **c** The heteronuclear multiple-bond correlation (HMBC) of PTIC in CDCl_3_. **d** The preferential conformation of PTIC. **e** The solution and **f** film UV–vis absorption of ID4F and NFRAs. **g** The UV–vis absorption of *I*_747 nm_/*I*_690 nm_ ratio for PTIC film annealed at 100 ^o^C for 320 min, with an inset of optical images

In order to understand the molecular conformation of the above NFRAs, we first conducted density functional theory (DFT) calculations (Fig. 2a and Supplementary Fig. 5). Within NFRAs, two potentially rotatable single C–C bonds (center single bond between alkyloxybenzene and thiophene, noted as PT rotamer; terminal single bond between thiophene and DFIC, noted as TIC rotamer) were present. The relaxed potential surface energy scans of possible rotamers were performed (Fig. 2a). The energy–torsion angle curves (*E*–*θ* curves) reveal that PT rotamer has a rotation energy barrier of 4.4 kJ mol^−1^, from the conformation with the lowest total energy (10^o^ PT rotamer featured with O–H interaction, O–H geometry), through 90° PT rotamer, to the conformation with the second lowest total energy (170° PT rotamer featured with O–S interaction between alkyloxybenzene and thiophene, O–S geometry). Note that O–H geometry has A slightly lower total energy than that of O–S geometry (Fig. 2a and Supplementary Fig. 5). It suggests that O–H geometry of NFRAs is the energetically preferential conformation, which, however, can thermodynamically access O–S geometry if rotation barrier of PT rotamer is being overcome. In addition, the *E*–*θ* curves of the terminal C–C bond (TICH rotamer for PTICH, TIC rotamer for PTIC, and TICO rotamer for PTICO) were compared to indicate that substituted thiophene helps preventing the generation of a 180^o^ rotamer (one of the possible isomers for NFRAs). It is because the steric hindrance between the thiophene substituent and DFIC significantly increases energy barriers over 45 kJ mol^−1^ (as a comparison, TICH rotamer with unsubstituted thiophene shows only a barrier of 6.25 kJ mol^−1^ at torsion angle of 180°). Theoretical investigations suggest that NFRAs adapt O–H geometry as their preferential conformation, wherein the center single bond has a relatively small rotation barrier, whereas, the terminal single bond shows less rotation tendency (Fig. 2a).

To probe the noncovalent O–H intramolecular interaction, we further conducted temperature-dependent ^1^H-NMR for NFRAs (Fig. 2b). Interestingly, the chemical shift of thiophene proton (H_a_) in PTIC (in d_4_-dichlorobenzene) gradually moves from 7.83 to 7.70 ppm, upon increasing temperature from 30 to 120 °C. It is the characteristic for breaking hydrogen bonds between H_a_ and oxygen atoms on alkyloxybenzene of the PT core at the elevated temperature. The hydrogen bond is also supported from the calculated H_a_–O distance that is shorter than the sum of Van der Waals radius for H and O atoms^35^. The proton (H_b_) on the benzene core also slightly shifts from 7.57 to 7.50 ppm, owing to the rotation of H_b_ away from DFIC. Nevertheless, aliphatic protons (H_c_ and H_e_) have no obvious changes. Similar results are also observed from the temperature-dependent ^1^H-NMR of PTICH and PTICO (Supplementary Figs. 6–8).

From the ^13^C−^1^H correlation spectra of PTIC in CDCl_3_, the heteronuclear multiple-bond correlation (HMBC) exhibits a signal at (7.71, 154) of H_a_ and C_3_ correlation (Fig. 2c, Supplementary Fig. 9), suggesting the existence of a preferential hydrogen-bond-based conformation. Still, the coexistence of S–O geometry in solution should not exclude due to the low rotation energy barrier of PT rotamer. To sum up, PTIC analogously adapts O–H geometry as a preferential conformation, which may be still thermodynamically accessible to O–S geometry in solution.

SSNMR of PTIC was further conducted to probe the molecular stacking behaviors in solids (Supplementary Figs. 10–14 and Supplementary Table 2). The results suggest “end-to-end” packing of PTIC in solid (Fig. 1b), which is similar, but in closer stacking distance to those reported FREAs (Supplementary Fig. 15)^36,37^. This observation is in agreement with GIWAXS measurements (which will be discussed later). From these theoretical and experimental results, we reveal that the NFRAs that have O–H geometry as a preferential conformation, which may be rotatable in solution (due to the low-energy barrier of center single C–C bond), can be restrained into a relatively rigid and planar structure with dense “end-to-end” stacking in solid. From the material processing point of view, this conformational rearrangement from solution to solid would endow NFRA acceptors not only good solution processability, but also excellent film optoelectronic properties.

### Optoelectronic properties

The backbone conformation greatly influences optoelectronic properties of a molecule. NFRAs (PTICH, PTIC, and PTICO) have features of rotatable conformation in solution, while being restrained into a planar conformation upon stacking together in the film. The ultraviolet–visible (UV–vis) absorption spectra of three NFRAs and fused ID4F in solution and thin films are shown in Fig. 2e. In solution, PTICH, PTIC, and PTICO exhibit broad absorption peaks without apparent aggregation shoulders, with maximum absorption (*λ*_abs_^sol^) located at 637, 648, and 647 nm on the molar extinction coefficients (*ε*_abs_^sol^) of 0.92 × 10^5^ M^−1^ cm^−1^ (PTICH), 1.12 × 10^5^ M^−1^ cm^−1^ (PTIC), and 1.18 × 10^5^ M^−1^ cm^−1^ (PTICO), respectively. These extinction coefficients are apparently lower than those of ID4F (*ε*_abs_^sol^ of 1.75 × 10^5^ M^−1^ cm^−1^ at 671 nm) in solution (Table 1 and Fig. 2e). It is because that fused ID4F has a rigid and planar structure in solution, ensuring stronger intramolecular charge transfer (ICT) effect and aggregation than those of non-planar NFRAs in solution.

**Table 1:** Optical properties of ID4F and NFRAs

	*λ*_abs_^sol^ (nm)	*λ*_abs_^film^ (nm)	*λ*_onset_^film^ (nm)	*E*_g_^opt^ (eV)	*λ*_em_^sol^ (nm)	*λ*_em_^film^ (nm)	SS (nm)	
							Sol	Film
ID4F	671	707	752	1.64	725	758	54	51
PTICH	637	709	775	1.60	722	778	85	69
PTIC	650	747	810	1.53	725	795	75	48
PTICO	647	746	794	1.56	718	795	71	49

Interestingly, the maximum absorption peaks of films (*λ*_abs_^film^) are observed at 709, 747, and 746 nm with strong aggregation shoulders for PTICH, PTIC, and PTICO, respectively (Fig. 2f). The *λ*_abs_^film^ of NFRAs displays a large redshift over 70 nm from solution to the film, whereas ID4F has only a 36-nm redshift. The extinction coefficients of films (*ε*_abs_^film^) of two NRFAs (PTIC, 0.99 × 10^5^ cm^−1^ and PTICO, 1.01 × 10^5^ cm^−1^) are almost in the same level of ID4F (1.06 × 10^5^ cm^–1^). These indicate that NFRAs in films not only form dense J-aggregation, but also adapt planar conformation with enhanced ICTs, resulting in more bathochromic and hyperchromic shifts than ID4F. Besides, the PTIC shows excellent conformational and morphological stabilities in the film, as indicated from temperature-dependent absorption measurements (Fig. 2g). When annealed from 25 to 200 °C, or at 100 ^o^C up to 360 min, both the intensity ratios (*I*_747 nm_/*I*_690 nm_) and absorption edge remain the same with no apparent changes (Fig. 2g, Supplementary Figs. 16 and 17). These results coincide with the differential scanning calorimetry (DSC) of acceptors without apparent thermal transitions below 200 °C (Supplementary Fig. 18). On the contrary, in chlorobenzene (CB) solution, the *λ*_abs_^sol^ of PTIC is blue-shifted from 650 to 639 nm upon increasing temperature from room temperature to 70 °C, which may be attributed to flexible and rotational conformation in the solution (Supplementary Fig. 19).

The photoluminescence (PL) spectra of ID4F and NFRAs were also measured in both solution and film (Supplementary Fig. 20 and Table 1). The emission of PTIC shows the dependence of solvent polarity, attributing the ICT effect of NFRAs upon excitation (Supplementary Fig. 21). The Stokes shift (SS) can be calculated from the difference between maximum absorption and emission, which links to the reorganization energy of a molecule between ground and excited-state transition. In solution, the SS values of PTICH, PTIC, and PTICO are 85, 75, and 71 nm, which are all apparently larger than 54 nm of ID4F, indicating that the nonradiative transition of NFRAs consumes more energies than that of FREA ID4F. Interestingly, in the film, the SS values of NFRAs are largely reduced (48 nm for PTIC and 49 nm of PTICO), suggesting that NFRAs in the film have less (at least similar) nonradiative energy losses than those of ID4F (51 nm), which are consistent with the calculated reorganization energy values (Supplementary Fig. 22). This is in agreement with the quantum efficiencies of non-fused acceptors in the solid state, which are apparently higher than those in solution; attributing to molecular packing in solid helps rigidifying the molecular structure and suppressing nonradiative decay, over those of flexible structures in solution.

**Fig. 3:**
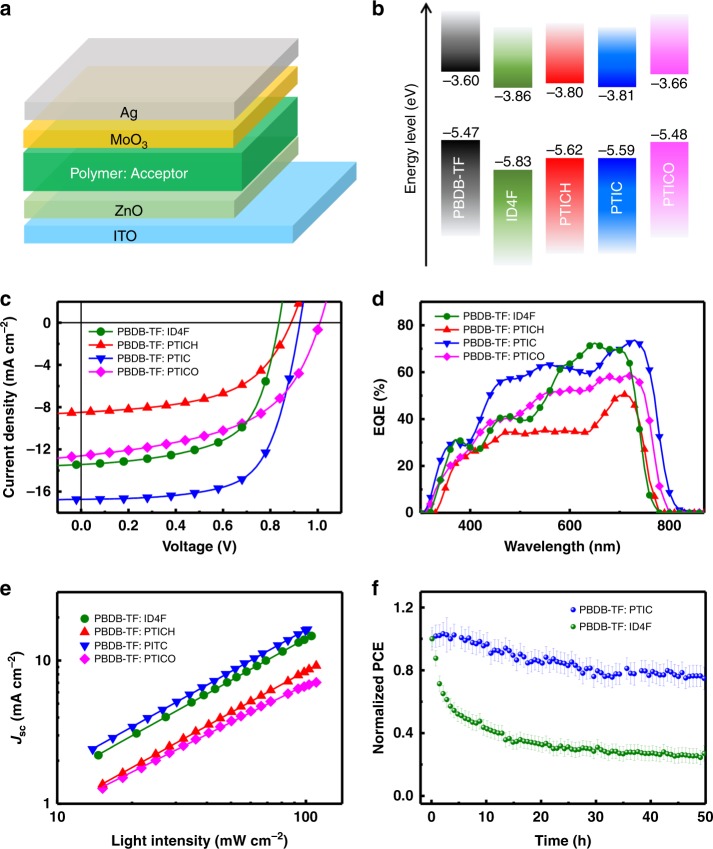
Energy levels and photovoltaic performance. **a** Inverted OSC device architecture. **b** Energy levels for different active components. **c** The *J*–*V* characteristics and **d** EQE spectra of OSCs under AM 1.5 G illumination (100 mW cm^–2^). **e** *J*_SC_ versus light intensity of the OSCs. **f** Stabilities of encapsulated devices under continuous illumination of a metal halide lamp without UV filtration. The error bars represent the standard deviation from four devices

The energy levels of these acceptors were estimated from cyclic voltammetry (CV) and ultraviolet photoelectron spectrometer (UPS) (Fig. 3b and Supplementary Table 3). NFRAs possess relatively upshifted energy levels over those of ID4F. They energetically match with the PBDB-TF polymer donor, except for PTICO, showing a small highest-occupied molecular orbital (HOMO) and the lowest-unoccupied molecular orbital offsets (0.01 and 0.06 V, respectively) with PBDB-TF (Supplementary Fig. 23).

### Photovoltaic properties

To investigate the influence of NFRA structural factors to their photovoltaic performance, we have fabricated inverted OSCs with an architecture of indium tin oxide (ITO)/ZnO/active layer/MoO_3_/Ag (Fig. 3a), wherein the active layer employs the PBDB-TF polymer donor and each acceptor (Fig. 3b), respectively. Note that all devices are fabricated from single-solvent processing without an additive, which may be feasible and easy for scale-up fabrication. All NFRA-based blends are cast with CB, and the ID4F blend is fabricated with CHCl_3_ due to its solubility preference. Their current density–voltage (*J*–*V*) characteristics are shown in Fig. 3c, and device performances are summarized in Table 2. The ID4F-based device exhibited the PCE of 6.88%, with the *J*_SC_ of 13.42 mA cm^−2^, *V*_OC_ of 0.84 V, and FF of 0.61. NFRA-based devices generally exhibit higher *V*_OC_ values (0.92 V for PTICH, 0.93 V for PTIC, and 1.01 V for PTICO) with mitigated voltage losses (0.68 V for PTICH, 0.58 V for PTIC, and 0.55 V for PTICO), comparing with those of fused ID4F (voltage losses of 0.80 V).

**Table 2:** The device parameters of OSCs with different acceptors under AM 1.5 G illumination (100 mW cm^–2^). The resistances were extracted from the *J*–*V* curves under light illumination

	*J*_SC_ (mA cm^–2^)	*V*_OC_ (V)	FF	PCE^a^ (%)	*V*_loss_ (V)	*R*_s_ (Ω cm^2^)	*R*_sh_ (Ω cm^2^)
ID4F	13.42 (13.11 ± 0.41)	0.84 (0.84 ± 0.01)	0.61 (0.60 ± 0.02)	6.88 (6.60 ± 0.25)	0.80	11.12	2630
PTICH	8.22 (7.96 ± 0.38)	0.92 (0.92 ± 0.01)	0.54 (0.53 ± 0.02)	4.08 (3.67 ± 0.40)	0.68	18.25	2057
PTIC	16.73 (16.50 ± 0.37)	0.93 (0.93 ± 0.01)	0.66 (0.65 ± 0.02)	10.27 (10.07 ± 0.19)	0.58	9.14	3091
PTICO	12.60 (12.03 ± 0.65)	1.01 (1.01 ± 0.01)	0.52 (0.49 ± 0.04)	6.62 (6.28 ± 0.34)	0.55	10.53	2800

^a^The values in the parentheses are the average PCEs from 20 devices

Encouragingly, PTIC-based device shows the decent performance with PCE of 10.27%, FF of 0.66, and *J*_SC_ of 16.73 mA cm^−2^, and optimal series resistance (*R*_s_), as well as shunt resistance (*R*_sh_), despite that PTICH-based and PTICO-based devices possess relatively low *J*_SC_ and FF values. When a non-halogenated solvent, such as *o*-xylene is employed, the PBDB-TF: PTIC-based device can still reach 9.53% PCE (Supplementary Fig. 24). The external quantum efficiency (EQE) spectra of the PBDB-TF: PTIC blend display a broadened photoresponse from 310 to 820 nm (Fig. 3d). The calculated *J*_SC_ values from the integration of EQE curves are 15.93 mA cm^−2^ (PBDB-TF: PTIC), 12.80 mA cm^−2^ (PBDB-TF: ID4F), 8.19 mA cm^−2^ (PBDB-TF: PTICH), and 12.18 mA cm^−2^ (PBDB-TF: PTICO), respectively, consistent with those from *J*–*V* measurements.

It is interesting to note that the overall EQE and photocurrent of the PTIC-based device are superior to other devices in these studies. To understand the possible reasons, electron mobilities of blends are measured by the space charge-limited current (SCLC) method, revealing similar levels of 3.4 × 10^–5^ cm^2^ V^−1^ S^−1^ (ID4F), 1.1 × 10^–4^ cm^2^ V^−1^ S^−1^ (PTICH), 4.3 × 10^–5^ cm^2^ V^−1^ S^−1^ (PTIC), and 1.7 × 10^−5^ cm^2^ V^−1^ S^−1^ (PTICO), respectively. The corresponding hole mobilities are 2.5 × 10^−4^ cm^2^ V^−1^ S^−1^ (ID4F), 2.3 × 10^−4^ cm^2^ V^−1^ S^−1^ (PTICH), 1.2 × 10^−4^ cm^2^ V^−1^ S^−1^ (PTIC), and 1.7 × 10^−4^ cm^2^ V^−1^ S^−1^ (PTICO). The ratios of charge carrier mobilities (electron mobility/hole mobility) were 0.13, 0.47, 0.37, and 0.10 (Supplementary Fig. 25). The charge recombination of devices was further investigated via tracking the light intensity (*P*_light_)-dependent *J–V* characteristics^26^. As shown in Fig. 3e, the slopes for *J*_SC_–*P*_light_ were 0.97, 0.96, 0.98, and 0.89 for ID4F-based, PTICH-based, PTIC-based, and PTICO-based devices, suggesting that all devices have good dissociation probabilities at short-circuit condition with slopes close to 1, except for PBDB-TF: PTICO-based device (with a smaller slope of 0.89). It can be ascribed to the small energetic offsets between PTICO and polymer, perhaps hindering the exciton disassociation; thus there is a monomolecular recombination of the related OSCs, resulting in the moderate *J*_SC_ (12.60 mA cm^−2^) and PCE (6.62%). The dependence of *V*_OC_ on the *P*_light_ reveals that the slopes of devices, are close to 1 *kT q*^−1^ (Supplementary Fig. 26), indicating that open-circuit conduction devices have mainly a bimolecular recombination without trap-assisted Shockley–Read–Hall (SRH) recombination^38^.

In light of the decent performance obtained from simple PTIC NFRA, we studied the stabilities of devices (PBDB-TF: PTIC and PBDB-TF: ID4F) under continuous 1-sun-equivalent illumination. Note that a metal halide lamp without UV filtration is employed as a light source and its light density is monitored with a silicon cell. Surprisingly, PBDB-TF: PTIC-based device allows maintaining about 70% of its initial PCE value for 50-h illumination, despite the harsh light source (containing a portion of high-energy UV photons) employed in studies. This is much better than that of PBDB-TF: ID4F-based devices (the remaining 25% of its initial value under the same condition). Four devices are used for standard deviation (Fig. 3f). To understand the intrinsic photostability of acceptors themselves, we have further compared the neat PTIC and ID4F films under constant 1-sun-equivalent illumination, revealing that PTIC is more stable than that of ID4F. As shown in Supplementary Fig. 27, neat PTIC film remained as green color and there was a relatively steady absorption at 747 nm, for 32-h constant illumination. However, the fused ID4F and the original green film were faded to nearly transparent after 16-h illumination, indicating the breaking of a conjugated system. The thermal stress stability of PTIC-based and ID4F-based OSCs was investigated, and remains insensitive to thermal treatments at 100 °C for 32 h (Supplementary Fig. 28). The stability difference may stem from the structural factors of NFRA without extension-fused rings and tetrahedron *sp*^3^ bridge carbon of FREA, as well as the capability of dense stacking in films, which helps gaining additional photostabilities of NFRA-based OSCs.

### Blend morphology

The top-surface morphologies of NFRA blend films were measured by atomic force microscopy (AFM) (Supplementary Fig. 29). It reveals that PBDB-TF: PTICH film shows high surface roughness (*R*_q_) of 21.6 nm, indicating the strong aggregation of such blend and non-ideal morphology. For PTIC-based and PTICO-based blends, the film surfaces are relatively smooth with an *R*_q_ of 5.83 nm for PTIC and 7.73 nm for PTICO.

**Fig. 4:**
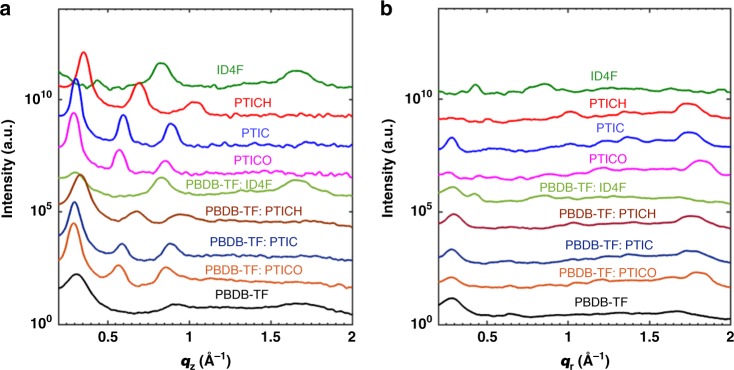
Morphologies of neat and blend films. The GIWAXS intensity profiles along the out-of-plane (**a**) and in-plane (**b**) directions

GIWAXS measurements were performed to study the molecular packing and crystallinity of the pure and blend films^25,37^. The intensity profiles in the out-of-plane and in-plane direction and 2D scattering patterns are shown in Fig. 4 and Supplementary Fig. 30, respectively. The peak positions and *d*-spacing are summarized in Supplementary Table 4. Pure ID4F film exhibits face-on orientation indicated by the π–π peak located at *q*_z_ = 1.66 Å^−1^ (*d* = 3.79 Å), similar to most FREAs. The additional peak at *q*_z_ = 0.825 Å^−1^ corresponds to a layer spacing twice of the π–π stacking distance, which is likely to originate from the spacing between the fused-ring core groups. Interestingly, all three NFRAs in neat films exhibit strong molecular stacking and high crystallinity, signified by sharp π–π peaks along the *q*_r_ axis and up to three orders of lamellar peaks along the *q*_z_ axis. The π–π stacking distances of PTICH, PTIC, and PTICO can be extracted as 3.63 Å for PTICH, 3.59 Å for PTIC, and 3.45 Å for PTICO. Unlike a fused ladder structure, ID4F possesses two upward and downward pointing chains onto a tetrahedron *sp*^3^ bridge carbon, NFRAs with alkoxy chains ensure shorter π−π stacking distances. This feature is also beneficial to reduce the Stoke shifts of NFRAs in films.

The scattering profiles of blend films reveal that NFRA-based blends exhibit stronger lamellar peaks than the ID4F blend does, indicating higher crystallinity and more ordered stacking for NFRA blends. Upon blending, PBDB-TF:ID4F has a slightly increased acceptor lamellar distance (15.0 Å). The peak at *q* = 0.305 Å^−1^ corresponds to the lamellar stacking of the donor. NFRAs in blends still exhibit similar lamellar and π–π stacking distances as in the neat films. It is evident that by removal of the covalent chemical bonding in fused-ring acceptors, NFRAs are given with a higher degree of freedom in solution, ultimately favoring the formation of higher crystallinity and tighter π−π stacking, compared with the fused-ring counterpart.

**Fig. 5:**
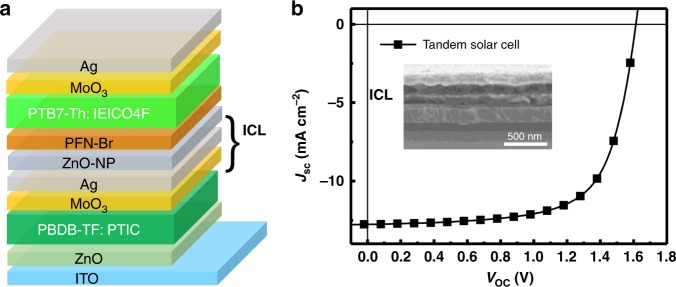
Efficient tandem solar cell. **a** Monolithic tandem architecture and **b** *J–V* curves of the tandem device under the illumination of AM 1.5 G (100 mW cm^−2^). The inset is the cross-sectional SEM image for tandem OSCs

As the PBDB-TF: PTIC BHJ showed efficient performance and a relatively high *V*_OC_ in single-junction OSCs, we further employed them into monolithic tandem OSCs with the architecture of ITO/ZnO/PBDB-TF: PTIC/MoO_3_/Ag/NP–ZnO/PFN-Br/PTB7-Th: IEICO-4F/MoO_3_/Ag (Fig. 5 and Supplementary Fig. 31). The interconnecting layer (ICL) is modified according to our previous approach^27^, wherein 0.5 nm of extra thin silver layer is employed for good charge recombination and light transmission. The tandem cells exhibited the *J*_SC_ of 12.76 mA cm^−2^, *V*_OC_ of 1.61 V, and FF of 0.68, resulting in the best PCE of 13.97% (PCE of 13.49% measured with aperture). It is highly encouraging that an exceptionally simple acceptor made from two-step synthesis allows leading to efficient and stable OSCs, which should be largely beneficial for their practical applications.

## Discussion

In this work, simple and effective electron acceptors with a fully unfused backbone have been developed through a two-step synthesis from single aromatic units. Through the combined experimental and theoretical investigations, we reveal that those NFRAs have structural features of rotatable conformation in solution, while being restrained into a relatively rigid and planar conformation upon stacking in the film, with the assistance of intramolecular non-covalent interaction. As a result, NFRAs exhibit not only feasible solution processability, but also excellent optoelectronic and stacking properties in films. These are beneficial factors to fabricate efficient OSCs through simple solution processing. Note that non-fused PTIC shows efficient performance of 10.27% PCE in single-junction OSCs and 13.97% PCE in tandem OSCs with a low synthetic complexity index. In addition, NFRA-based OSCs exhibit promising stabilities under continuous illumination. Overall, our studies reveal valuable insights on the structural design of simple and effective molecules with great practical values.

## Methods

### General synthesis of NFRAs

The non-fused core, 5,5′-(2,5-bis((2-hexyldecyl)oxy)-1,4-phenylene)bis(3-substituent-2-thenaldehyde) (i.e., PTH-CHO or PT-CHO or PTO-CHO) (0.20 mmol), DFIC (0.50 mmol), and a few drops of pyridine were dissolved in chloroform (CHCl_3_). The reaction mixture was refluxed overnight, and then concentrated in vacuo. The solid crude products were obtained through methanol precipitation, and washed with methanol and acetone, respectively. The final crystalline solid was obtained through the recrystallization from CHCl_3_ and methanol, and washed with a mixture of CHCl_3_/methanol (*v*/*v* = 1/1) to obtain pure product. The detailed synthesis is described in Supplementary Methods, and the final products were characterized by ^1^H-NMR, ^13^C-NMR, and mass spectra (Supplementary Figs. 35–44).

### Single-junction cell fabrication

Polymer solar cells were fabricated on glass substrates commercially pre-coated with a layer of ITO with the inverted structure of ITO/ZnO/PBDB-TF: Acceptor/MoO_3_/Ag. Prior to fabrication, the substrates were cleaned using detergent, deionized water, acetone, and isopropanol consecutively for every 15 min, and then treated in an ultraviolet ozone generator for 15 min. A thin layer of sol–gel ZnO was spin-coated onto clean ITO-coated glass substrates at 3500 rpm for 60 s and then annealed at 170 ^o^C for 20 min; then the substrates were transferred to a glovebox. The PBDB-TF: ID4F in CHCl_3_ (16 mg mL^−1^, 1:1.2 wt%) and PBDB-TF: NFRAs in CB (20 mg mL^−1^, 1:1.2 wt%) were then spin-coated on the ZnO layers. Then the BHJ layers were annealed at 100 °C under nitrogen atmosphere. The, the MoO_3_ layer (4 nm) was deposited on the active layers. Finally, silver (100 nm) was thermally evaporated through shadow masks to complete the device with an active area of 6 mm^2^.

### Tandem cell fabrication

The active layer for the front cell was first spin-coated atop of ZnO/ITO substrates from PBDB-TF: PTIC in CB (20 mg mL^−1^, 1:1.2 wt%) at 2000 rpm for 60 s, and annealed at 120 ℃ for 10 min. Then, MoO_3_ (4 nm) and ultrathin Ag (0.5 nm) were deposited via thermal evaporation. The deposition of the ultrathin Ag layer was defined by a mask with good alignment to the patterned ITO strip. Atop of an ultrathin Ag layer, ZnO–NP and PFN-Br in methanol were subsequently spin-coated to complete ICL fabrication. Afterward, the active layer for the rear cell was spin-coated from PTB7-Th: IEICO-4F in CB (5% CN) (30 mg mL^−1^, 1:1.5 wt%) at 3000 rpm for 60 s. The tandem device was completed by evaporating 4 nm of MoO_3_ and 100 nm of Ag. The active area of tandem cells is 6 mm^2^ (without aperture) and 3.75 mm^2^ (with aperture).

### *J*–*V* measurements

The current density–voltage (*J*–*V*) curves of OSCs were measured with Keithley 2400, under AM 1.5 G illumination at 100 mW cm^−2^ irradiation using a Enli SS-F5-3A solar simulator, and the light intensity was calibrated with a standard Si solar cell with a KG5 filter (made by Enli Technology Co., Ltd., Taiwan, and a calibrated report can be traced to NREL). The EQE spectrum was measured using a QE-R Solar Cell Spectral Response Measurement System (Enli Technology Co., Ltd., Taiwan). The EQE spectrum for the rear and front cells of tandem OSC was measured under the light bias obtained from 550-nm low-pass and 850-nm high-pass optical filters (upon exciting the front and rear cells), respectively. The mismatch factor (MM) values of the front cell and back cell are calculated to be 1.006 and 1.076, respectively, from the following equation:

1 $${\mathrm{{MM}}} = \frac{{\smallint E_{{\mathrm{ref}}}\left( \lambda \right)S_{{\mathrm{ref}}}\left( \lambda \right){\mathrm{d}}\lambda \cdot \smallint E_{{\mathrm{meas}}}\left( \lambda \right)S_{{\mathrm{sample}}}\left( \lambda \right){\mathrm{d}}\lambda }}{{\smallint E_{{\mathrm{meas}}}\left( \lambda \right)S_{{\mathrm{ref}}}\left( \lambda \right){\mathrm{d}}\lambda \cdot \smallint E_{{\mathrm{ref}}}\left( \lambda \right)S_{{\mathrm{sample}}}\left( \lambda \right){\mathrm{d}}\lambda }}$$

*E*_ref_(*λ*) is reference spectral irradiance of AM 1.5 G spectrum, *E*_meas_(*λ*) is the spectrum of the testing solar simulator; *S*_ref_(*λ*) and *S*_sample_(*λ*) are the spectrum response of a standard cell used to calibrate the simulator light intensity, and the spectral response of the sample solar cell under test, respectively.

### Device stability test

OSC devices are sealed with a cover glass by an UV-curing adhesive of epoxy resin LX803 (purchased from Solarmer Company). The stability of the encapsulated devices was measured in air under continuous 1 sun-equivalent illumination provided by a metal halide lamp (PHILIPS MSR 1200HR) without UV filtration (temperature: 45 ± 5 ℃). Light intensity was monitored by standard silicon solar cells.

## Supplementary information

Supplementary Information

Solar Cells Reporting Summary

## Data Availability

The data that support the findings of this study are available from the corresponding author upon reasonable request.
