# Supplementary material for: Simple non-fused electron acceptors for efficient and stable organic solar cells
Source: Nat Commun. 2019 May 14;10:2152. doi: 10.1038/s41467-019-10098-z (PMC6517432; doi:10.1038/s41467-019-10098-z)
Supplement: Supplementary file 1 — Supplementary Information [file 41467_2019_10098_MOESM1_ESM.pdf]

SUPPLEMENTARY INFORMATION

Simple Non-fused Electron Acceptors for Efficient Organic Solar Cells

Zhi-Peng Yu[1], Zhi-Xi Liu[1], Fang-Xiao Chen[1], Ran Qin[1], Tsz-Ki Lau[2], Jing-Lin Yin[3], Xueqian Kong[3], Xinhui Lu[2], Minmin Shi[1], Chang-Zhi Li[1] and Hongzheng Chen[1]

1. State Key Laboratory of Silicon Materials, MOE Key Laboratory of Macromolecular Synthesis and Functionalization, Department of Polymer Science and Engineering, Zhejiang University, Hangzhou 310027, P. R. China. E-mail: czli@zju.edu.cn

2. Department of Physics, The Chinese University of Hong Kong, New Territories, Hong Kong, China.

3. Department of Chemistry, Zhejiang University, Hangzhou 310027, China

Supplementary Figure

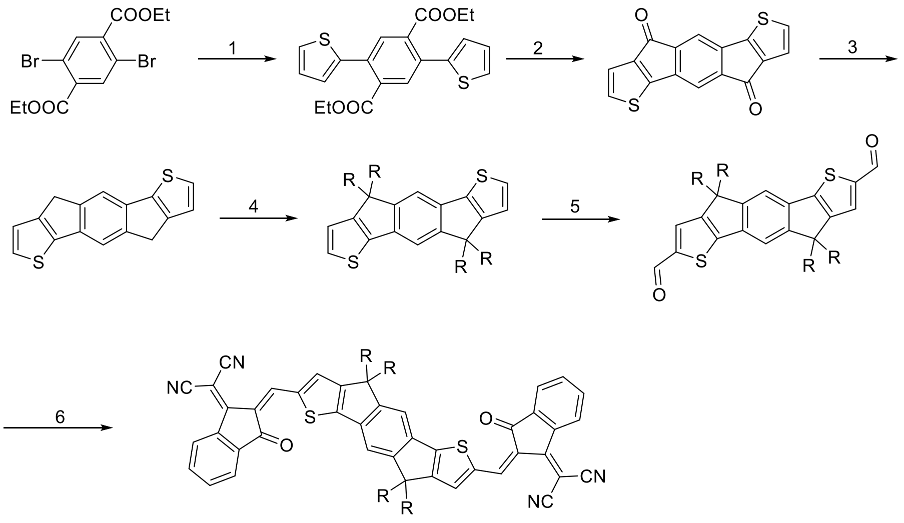

1. Stille/Suzuki Coupling; 2. Cyclization Reaction; 3. Reduction; 4. (a) n-BuLi, THF (b) RBr; 5. Vilsmeier-Haack Reaction; 6. Knoevenagel Reaction.

Supplementary Figure 1. General Synthesis of FREAs with alkyl side-chain.

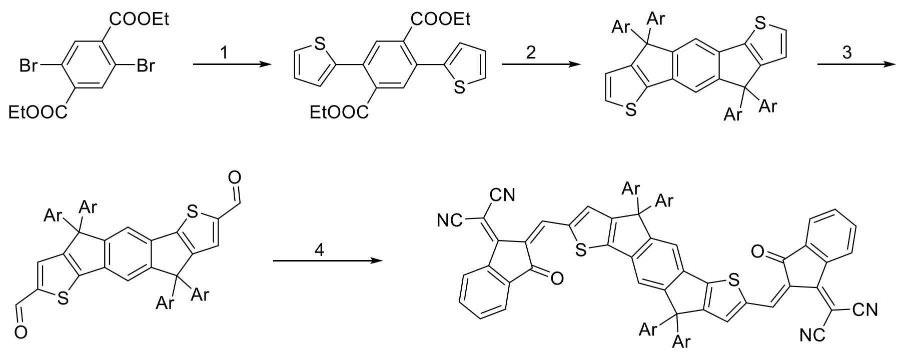

1. Stille/Suzuki Coupling; 2. Cyclization Reaction; 3. Vilsmeier-Haack Reaction;
4. Knoevenagel Reaction.

Supplementary Figure 2. General Synthesis of FREAs with aryl side-chain.

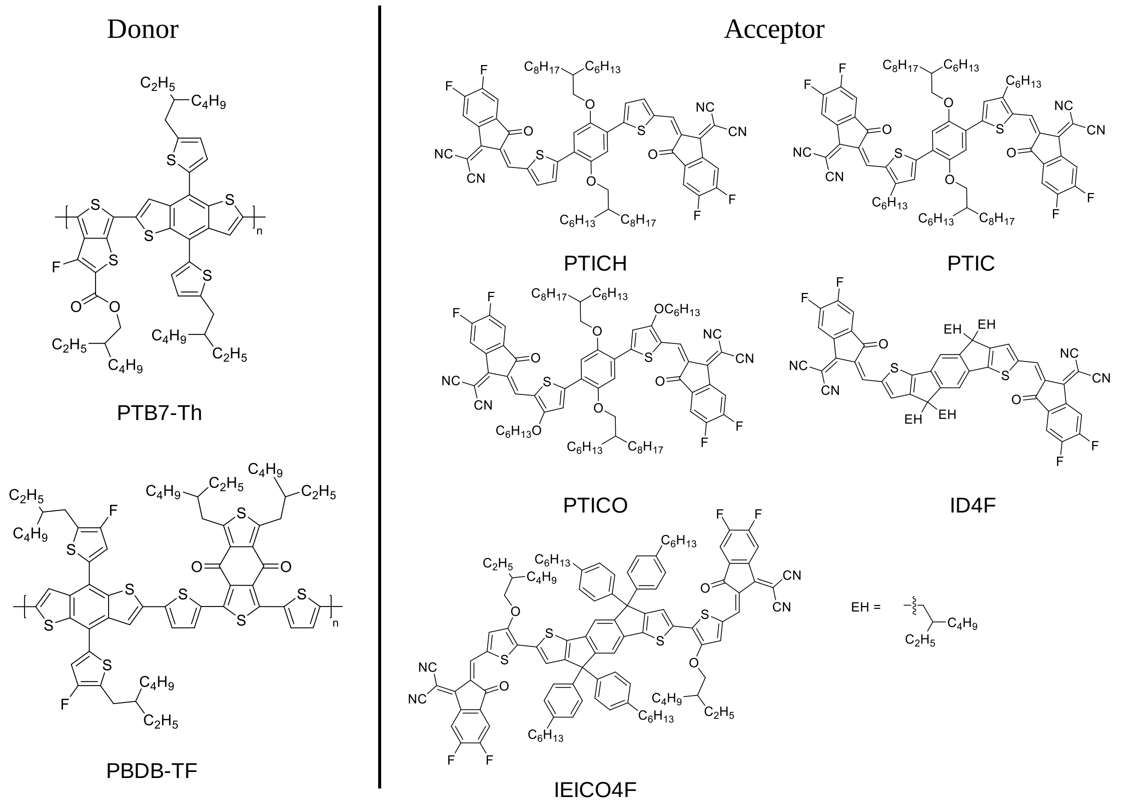

Supplementary Figure 3. Chemical structures of the studied materials in this work.

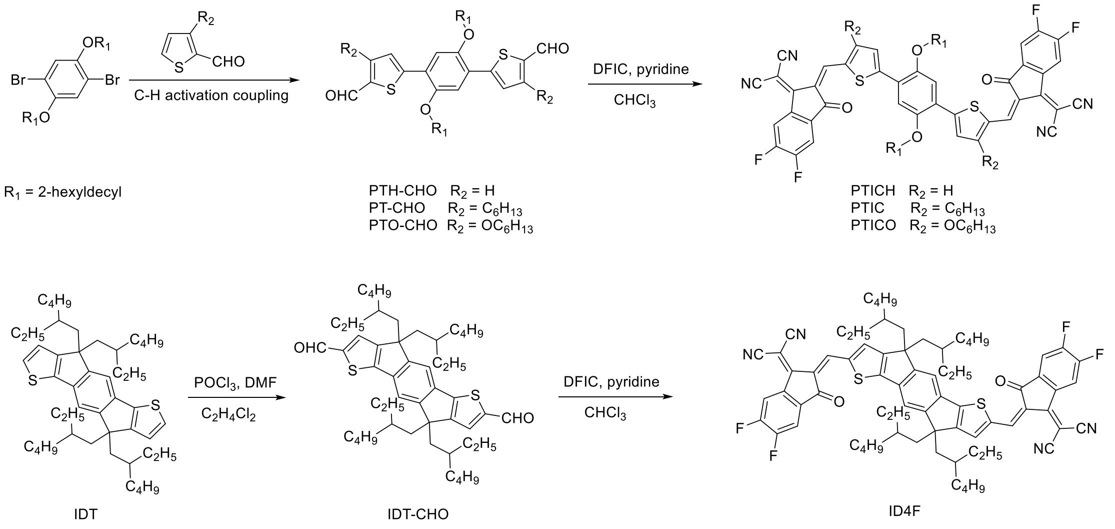

Supplementary Figure 4. The synthetic route for non-fused acceptors and fused-ID4F.

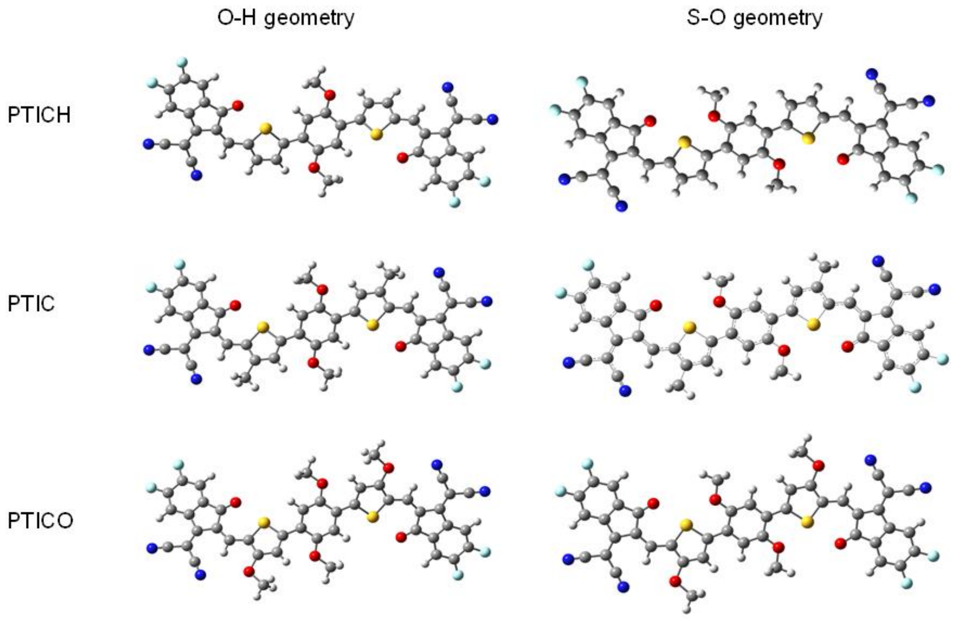

Supplementary Figure 5. The calculated results for the simplified **PTICH**, **PTIC** and **PTICO**. The O-H distance of O-H geometry are around 0.23 nm.

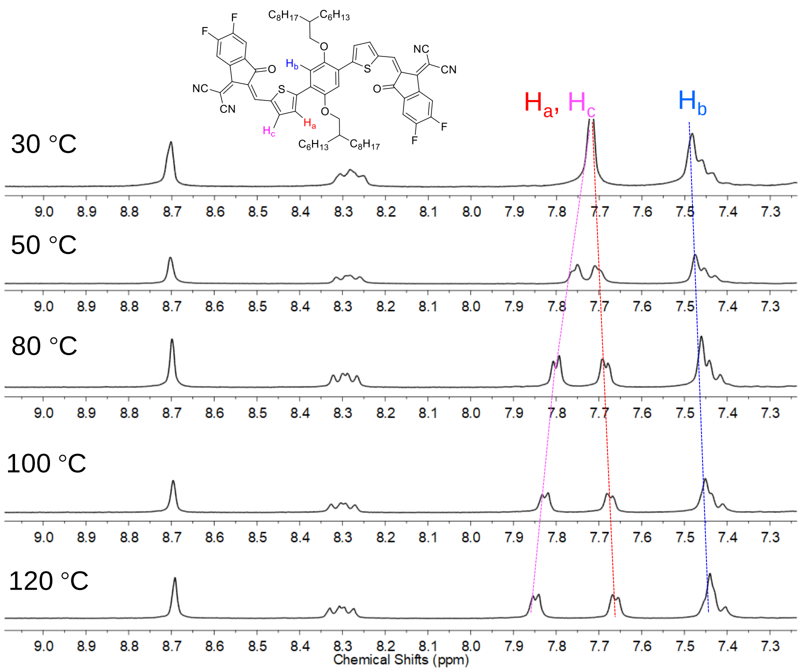

Supplementary Figure 6. The temperature-dependent ^{1}H-NMR of PTICH in d_4-$C_6D_4Cl_2$.

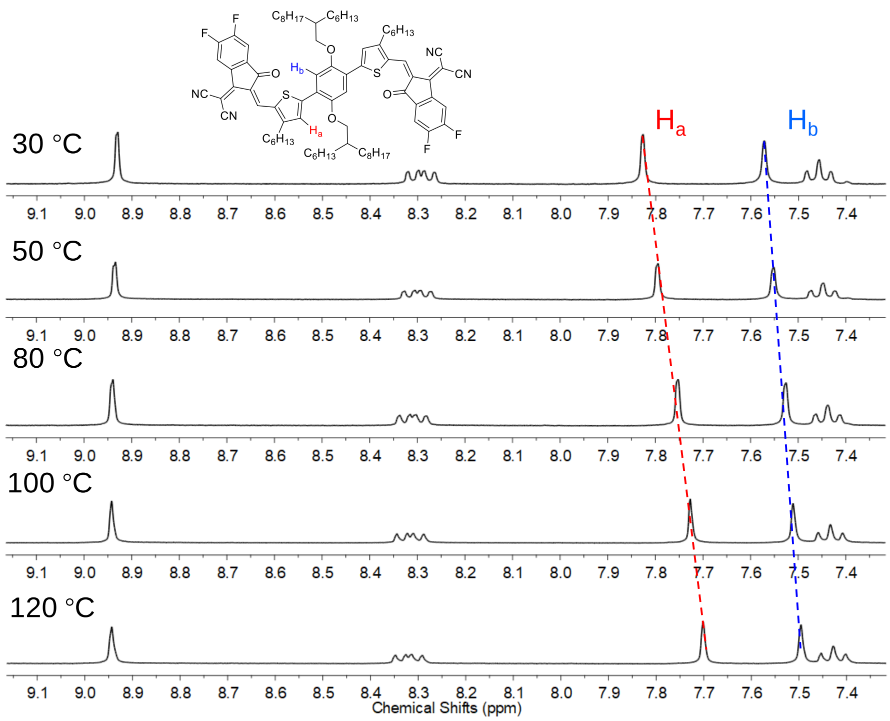

Supplementary Figure 7. The temperature-dependent ^{1}H-NMR of PTIC in d_4-$C_6D_4Cl_2$.

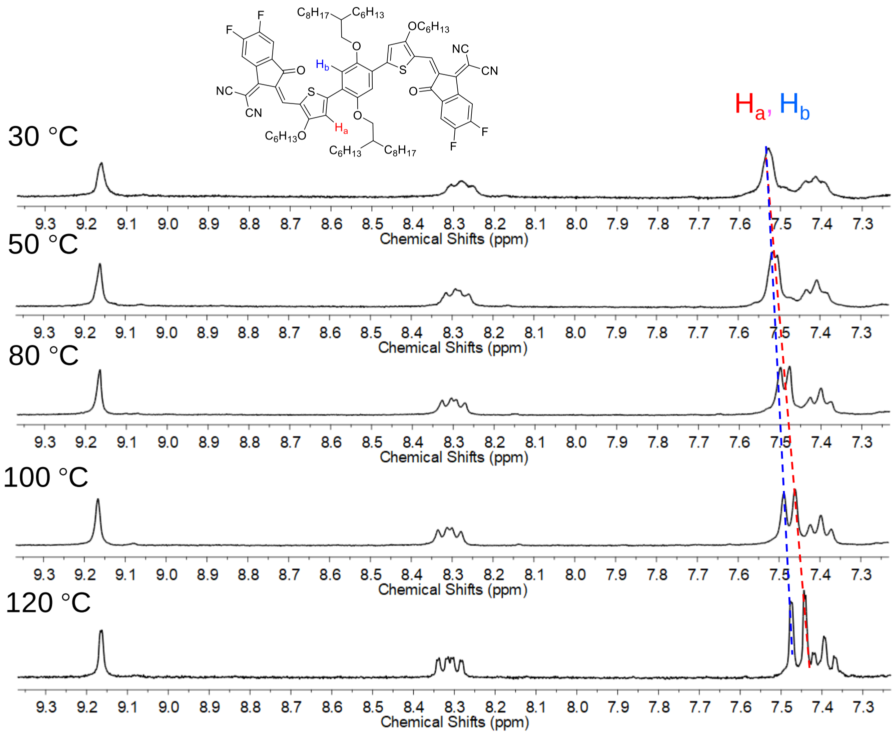

Supplementary Figure 8. The temperature-dependent ^{1}H-NMR of PTICO in d_4-$C_6D_4Cl_2$.

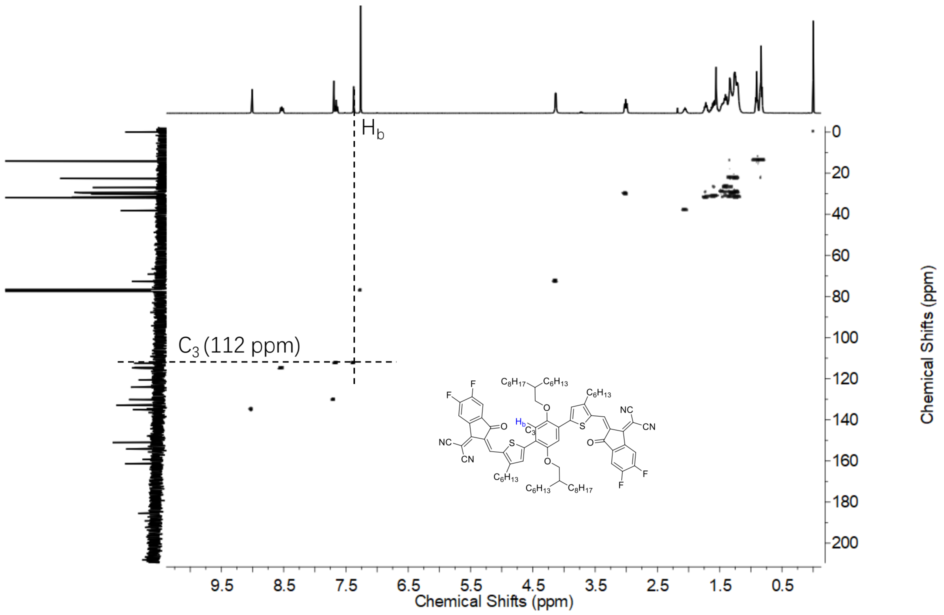

Supplementary Figure 9. The HSQC of PTIC in $CHCl_3$. The signal at 112 ppm in ^{13}C-NMR is attribute to C_1.

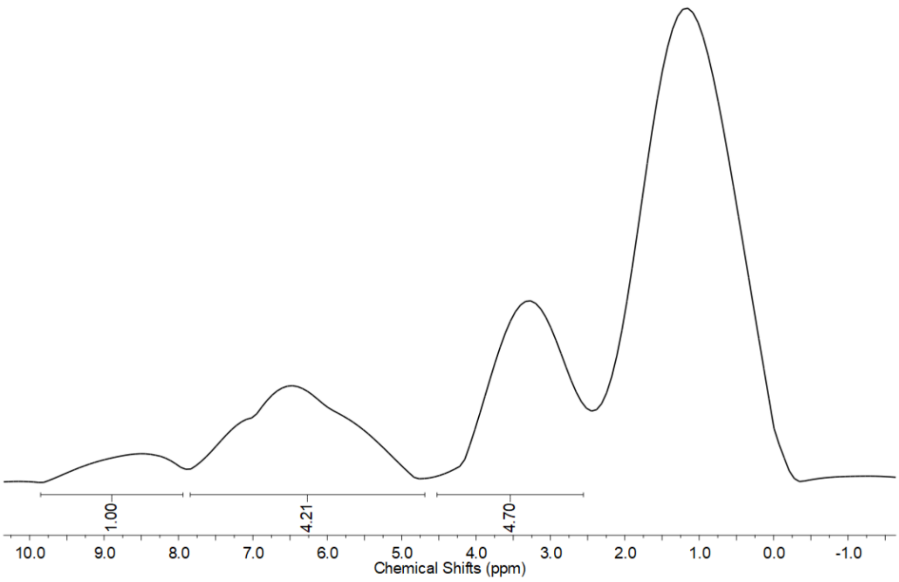

Supplementary Figure 10. The ^{1}H-SSNMR for PTIC.

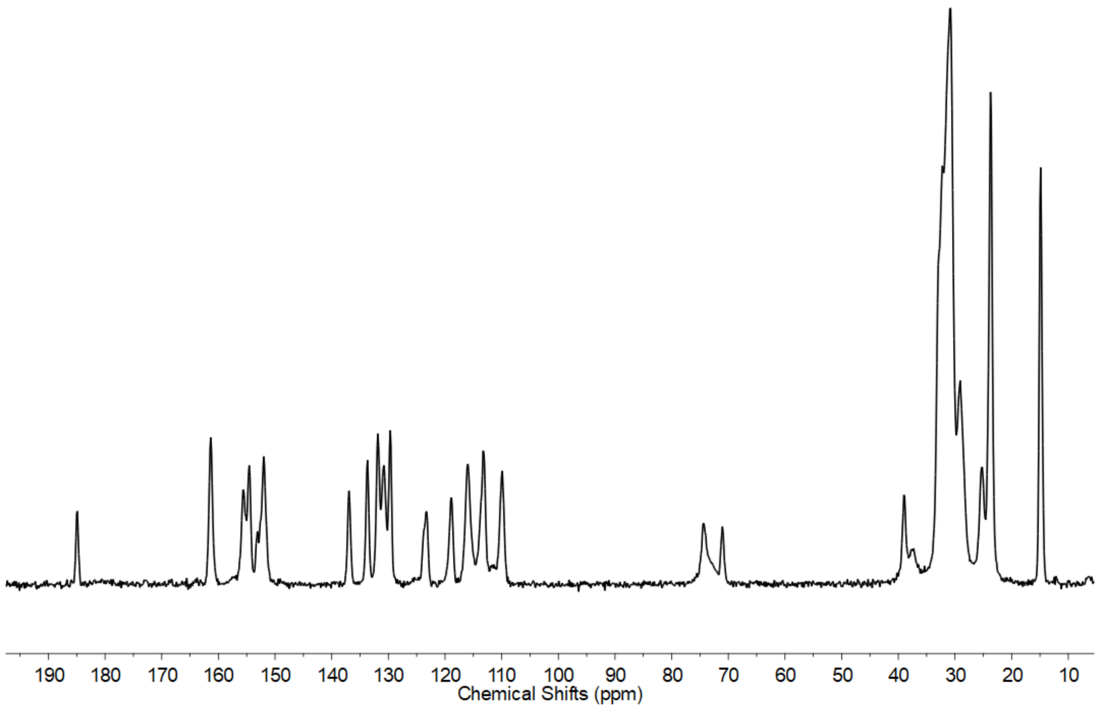

Supplementary Figure 11. The ^{13}C-SSNMR for PTIC.

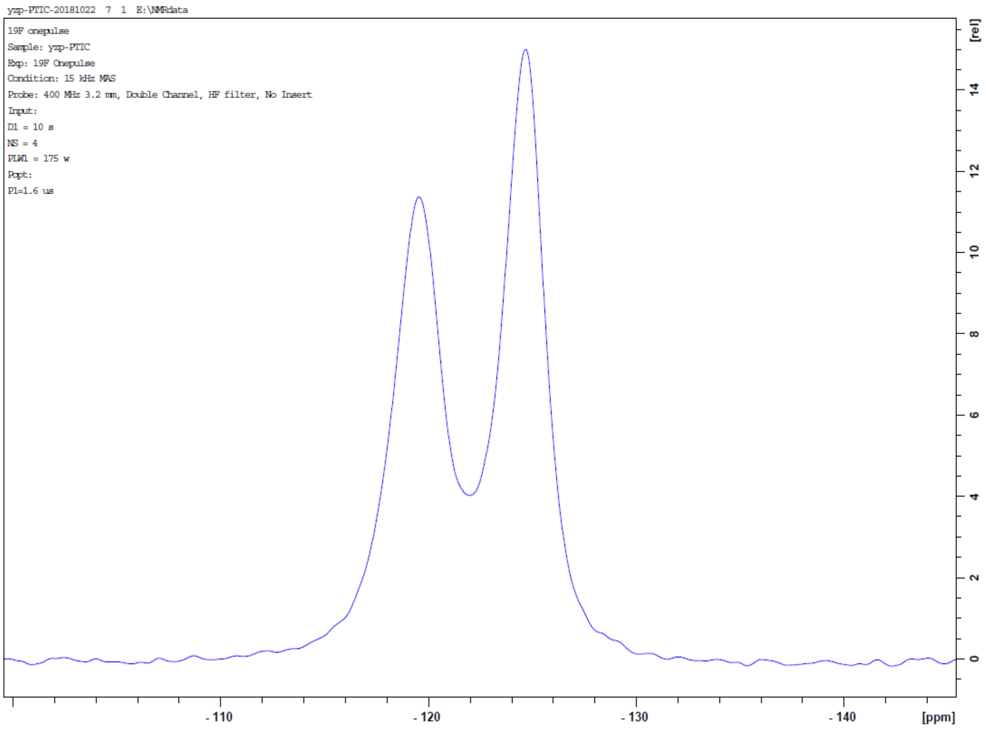

Supplementary Figure 12. The ^{19}F-SSNMR for PTIC.

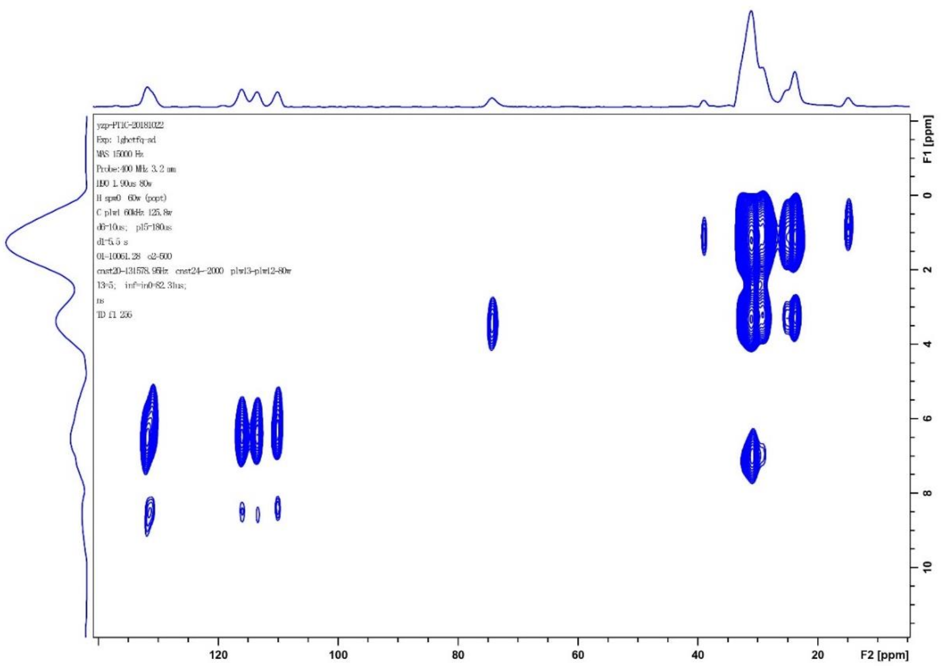

Supplementary Figure 13. The ^{13}C-^{1}H HETCOR NMR for PTIC with contact time of 180 ms and proton spin diffusion time of 10 μs. It indicates the aromatic carbons with chemical shifts of 130.9, 116.0, 113.3 and 110.0 ppm are chemically linked with protons (**Supplementary Table 2**).

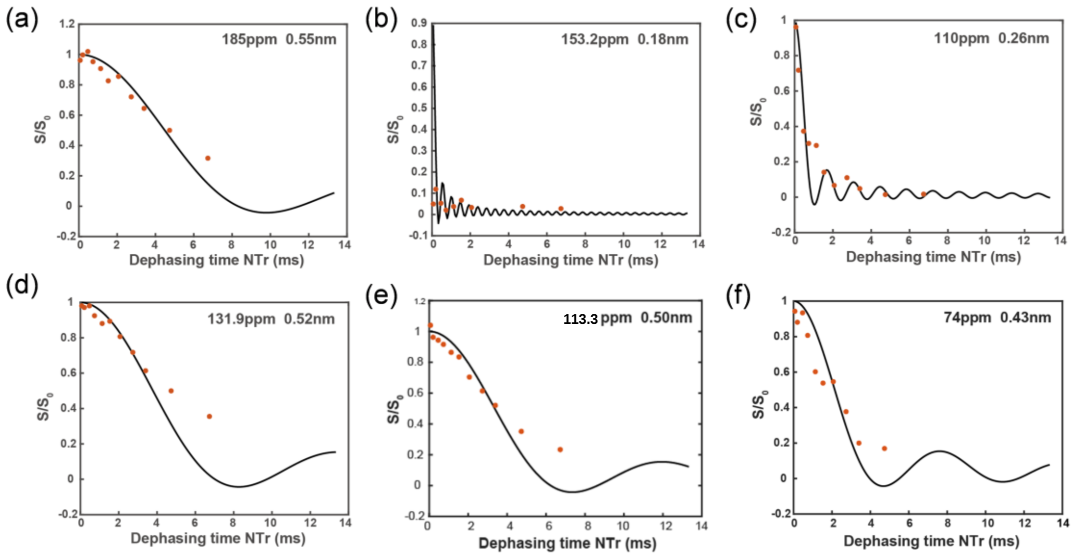

Supplementary Figure 14. The REDOR for PTIC. The data are listed in (**Supplementary Table 2**). As the carbon signal at 153.2 ppm is most sensitive with short distance of 0.18 nm to F-atom, attributing to the signal of $C_{37/38}$ (**Supplementary Figure 34**). Moreover, as the carbon with chemical shifts of 110 ppm is aromatic carbon linked with hydrogen directly (^{13}C-^{1}H HETCOR NMR), it is with the distance of 0.26 nm to F-atom[5], which can be assigned to $C_{36/39}$. Interesting, the distance of F-atom to C_1 and C_{21} are estimated be 0.50-0.52 nm, which are even smaller than their intramolecular distances. As C_1 and C_{21} belong to the aromatic carbons on PT core, these values reflect the intermolecular contact distance between DFIC and PT, indicating the “end-to-end” packing of NFRA molecules in solid.

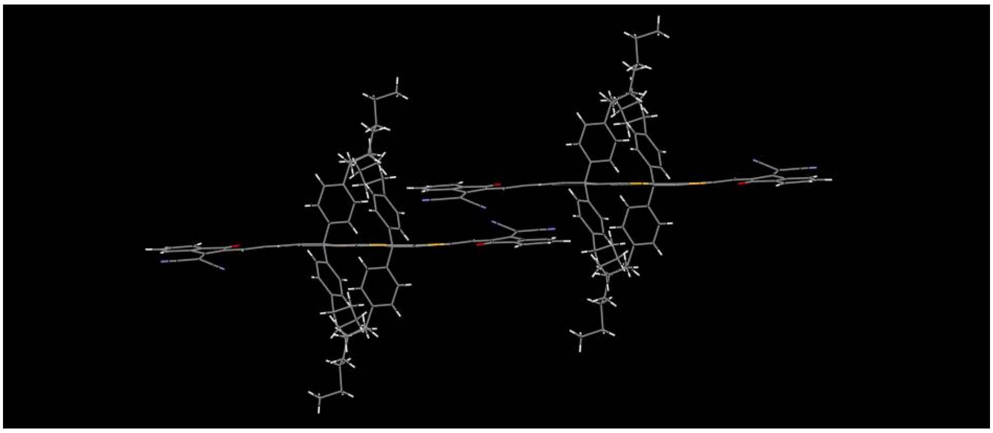

Supplementary Figure 15. The reported single crystal for 4TIC. [6]

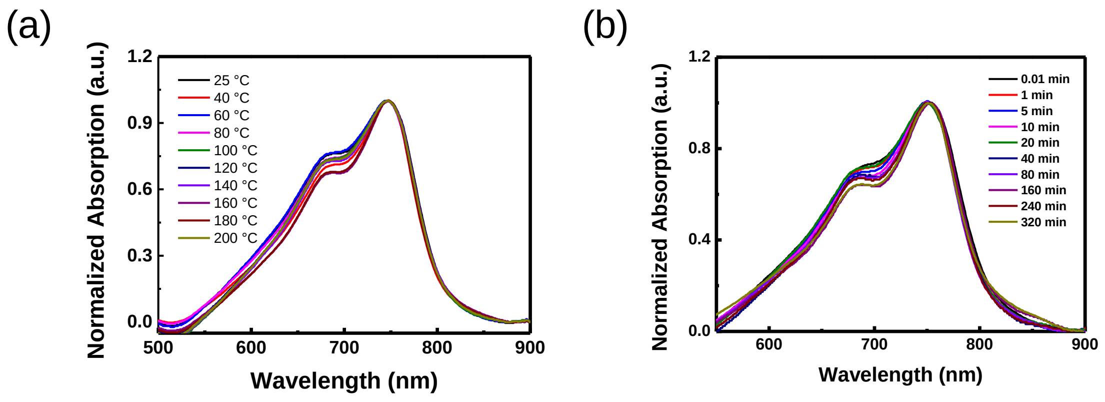

Supplementary Figure 16. The absorption of PTIC film with variety of temperature (a) and variety of time with temperature of 100 °C (b).

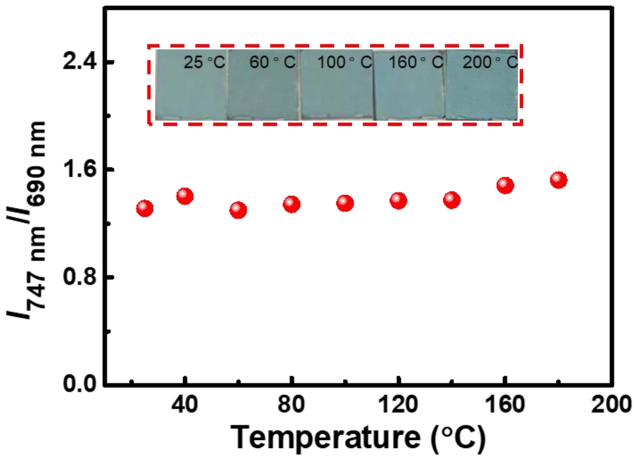

Supplementary Figure 17. The UV-vis absorption of $I_{747\ nm}/I_{690\ nm}$ ratio for PTIC films at varied temperature annealing (insert of optical images).

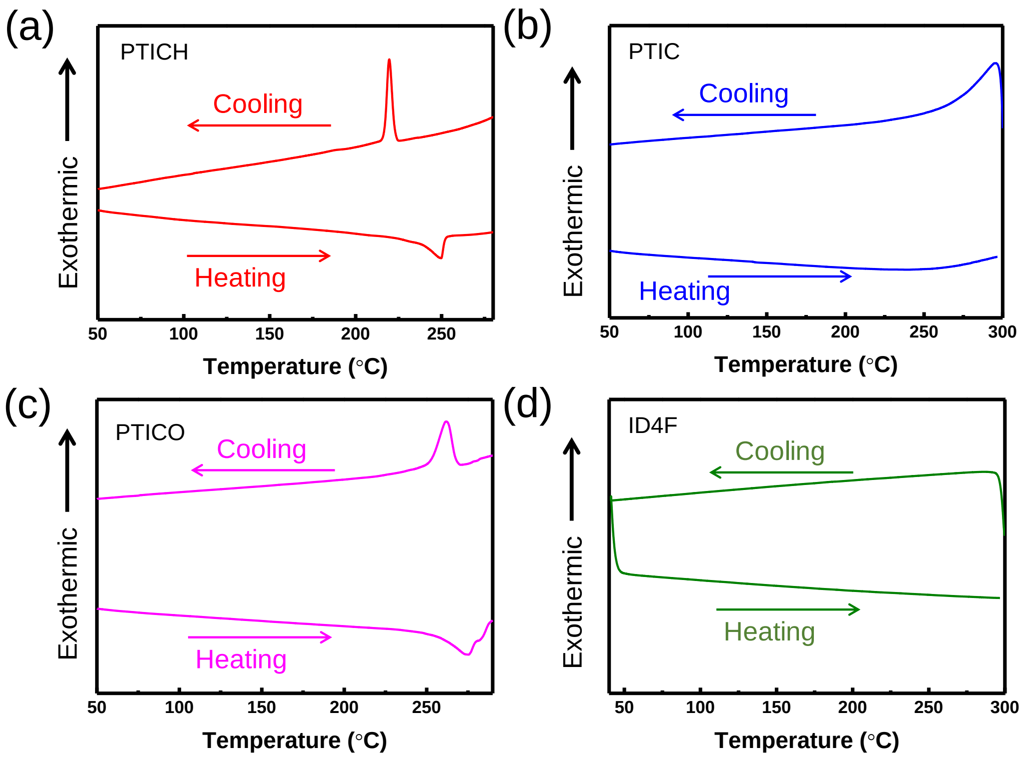

Supplementary Figure 18. The DSC curves of PTICH, PTIC, PTICO and ID4F recorded under N_2 atmosphere at a heating rate of 10 ℃ min^{-1}.

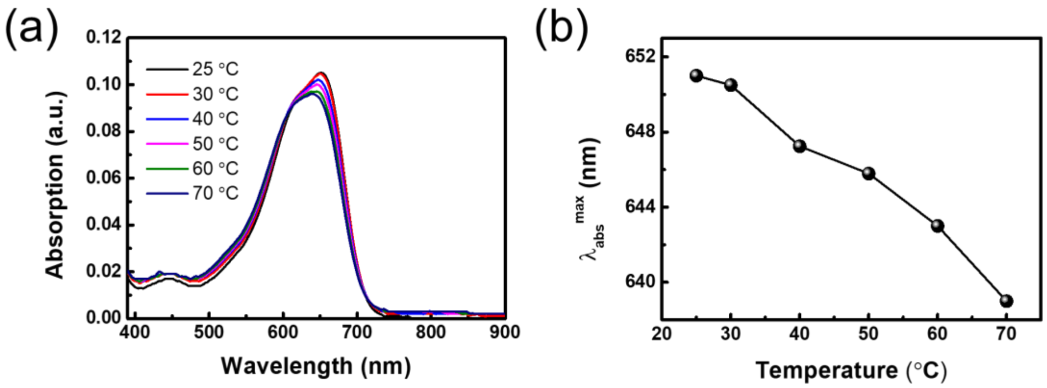

Supplementary Figure 19. The temperature-dependent UV-vis spectra of PTIC in chlorobenzene.

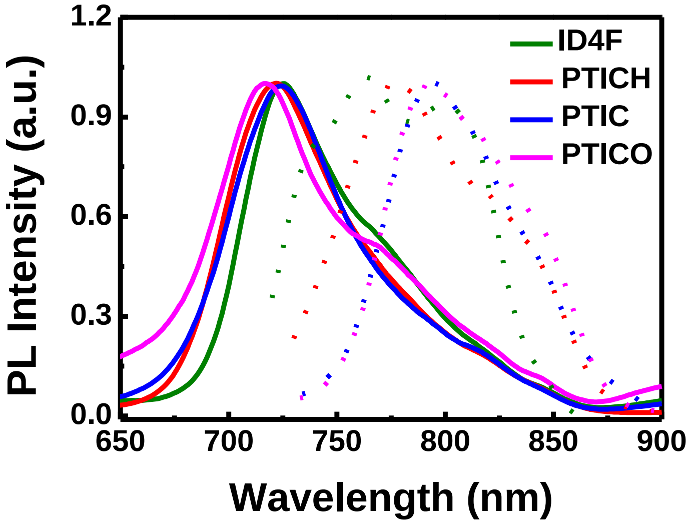

Supplementary Figure 20. The PL spectra of ID4F and NFRAs (solid line in chlorobenzene solution and dash line for films).

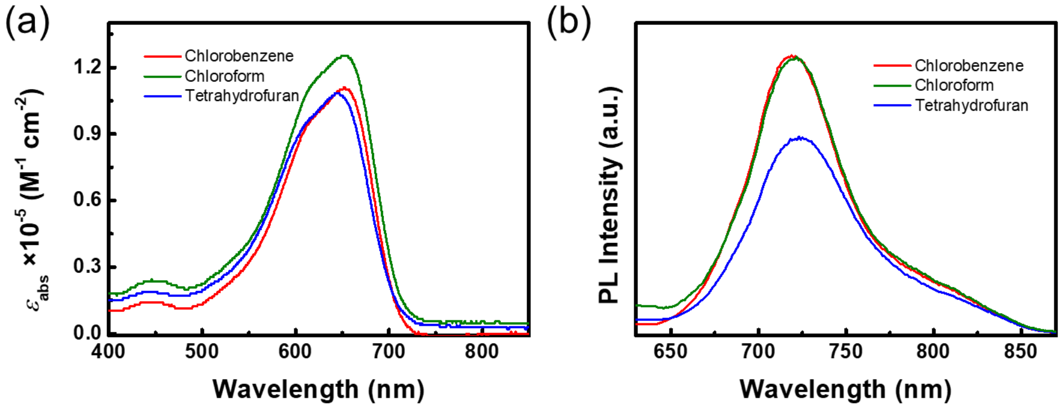

Supplementary Figure 21. The solvent-dependent (a) absorption and (b) emission of PTIC.

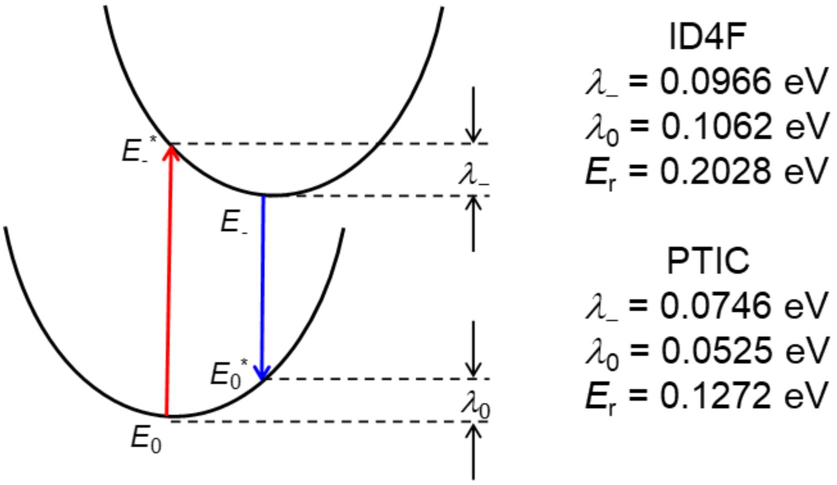

Supplementary Figure 22. The calculated results for reorganization energy.

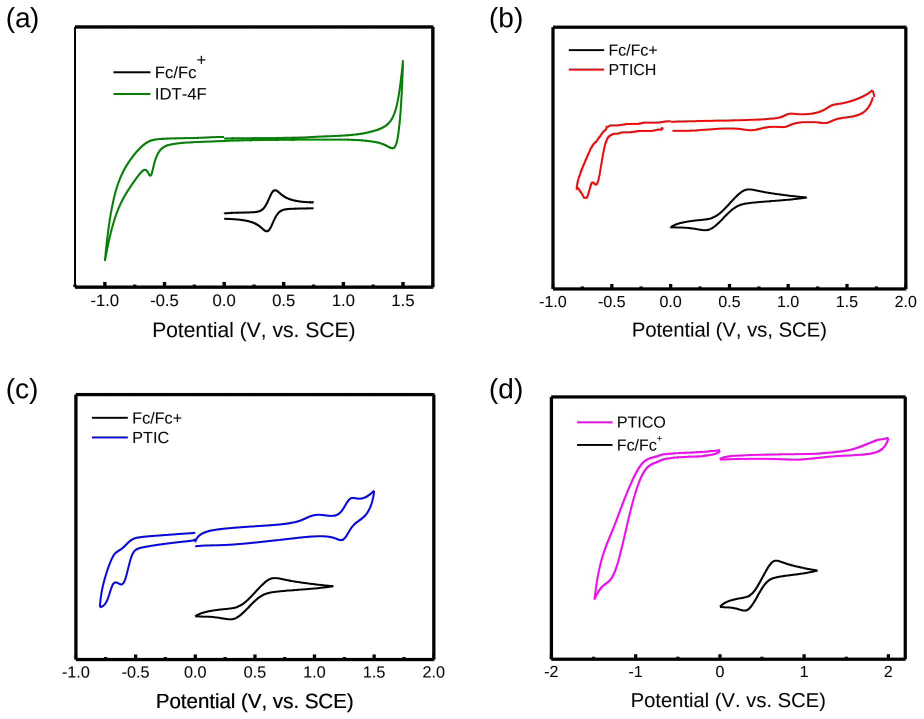

Supplementary Figure 23. The Cyclic Voltammetry plots of (a) ID4F, (b) PTICH, (c) PTIC and (d) PTICO in dichloromethane solution.

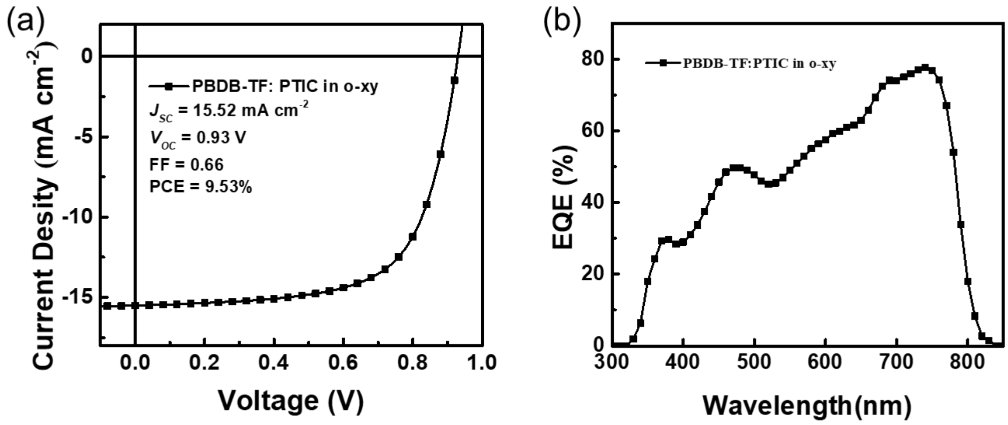

Supplementary Figure 24. (a) The *J–V* characteristics (b) and EQE spectra of PBDB-TF: PTIC based device fabricated from o-xylene solution under AM 1.5G illumination (100 mW cm^{-2}).

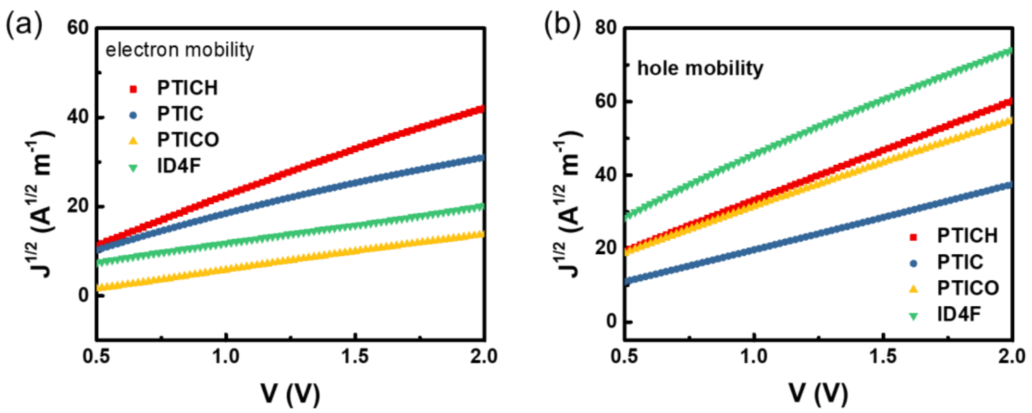

Supplementary Figure 25. The SCLC (a) hole mobility and (b) electron mobility of blend films based on NFRAs and fused-ID4F.

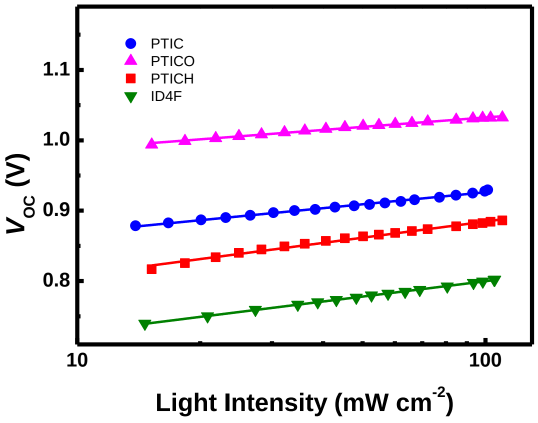

Supplementary Figure 26. The light-intensity (*P*) dependence of V_{oc} of the corresponding devices. The dependence of V_{OC} on the Plight are measured with the slopes of V_{oc} versus ln(P_{light}) of 1.22 kT e^{-1} (PBDB-TF: ID4F), 1.29 kT e^{-1} (PBDB-TF: PTICH), 1.01 kT e^{-1} (PBDB-TF: PTIC) and 1.02 kT e^{-1} (PBDB-TF: PTICO).

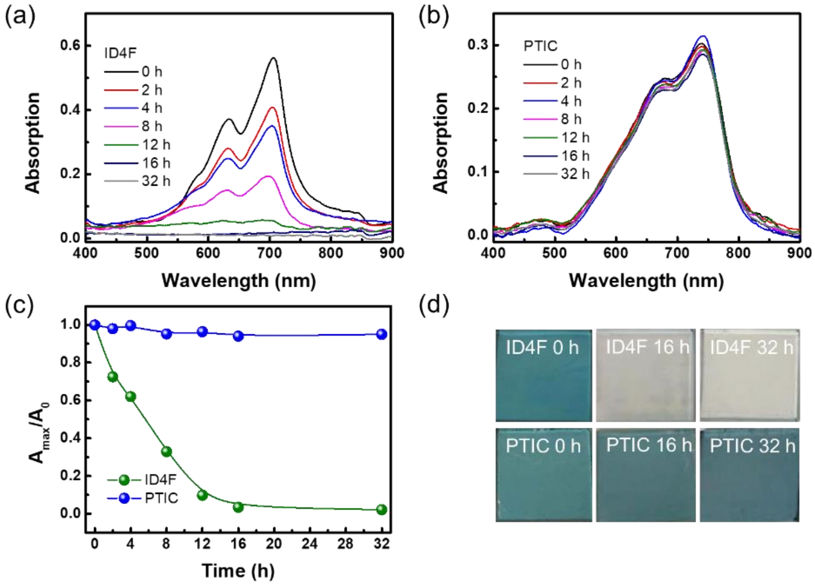

Supplementary Figure 27. The UV-vis spectra of (a) ID4F, (b) PTIC, (c) the relative ratio of absorption changes for PTIC and ID4F, and (d) the optical image of PTIC and ID4F under constant 1 sun equivalent illumination.

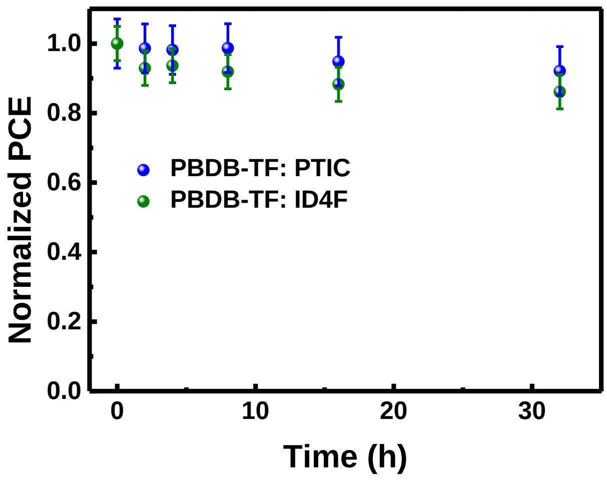

Supplementary Figure 28. The performance for the devices based on PBDB-TF: ID4F and PBDB-TF: PTIC blends after thermal treatment at 100 ℃ for various times. The error bars represent the standard deviation from four devices.

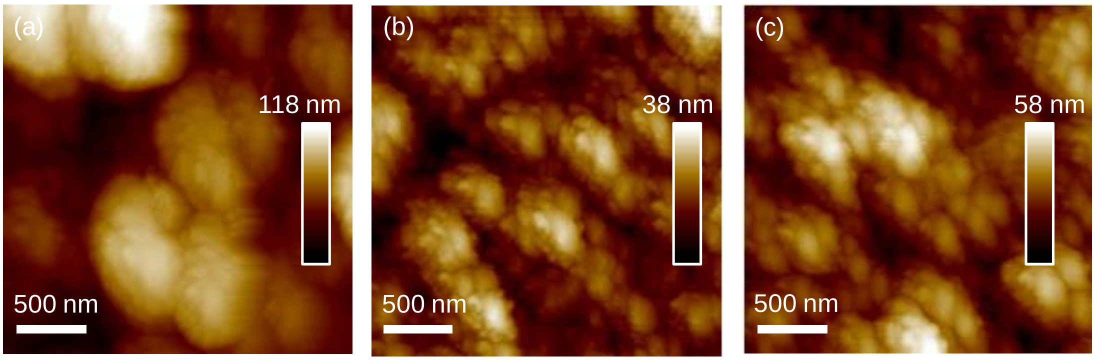

Supplementary Figure 29. The AFM of (a) PBDB-TF: PTICH film, (b) PBDB-TF: PTIC film and (c) PBDB-TF: PTICO film.

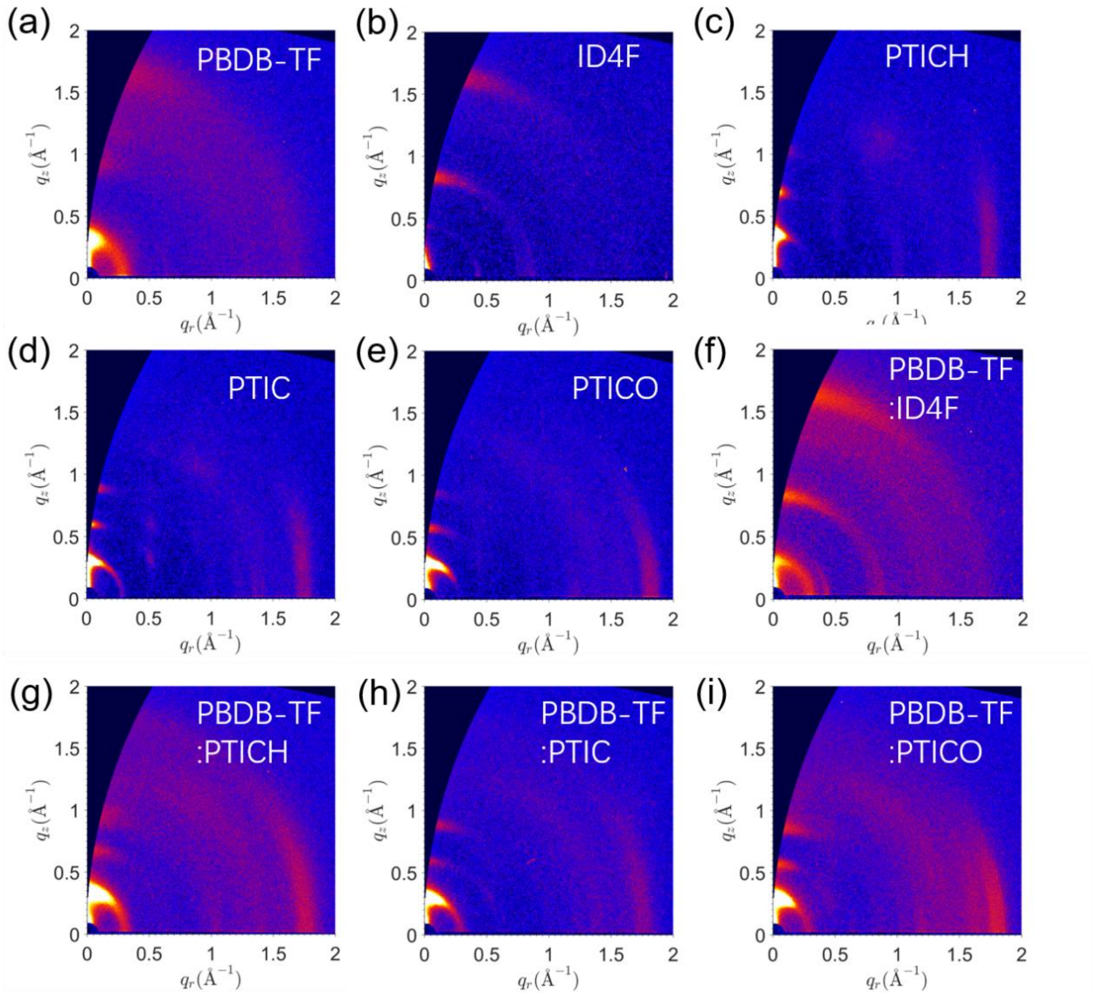

Supplementary Figure 30. The 2D-GIWAX for PBDB-TF, acceptors and blended films.

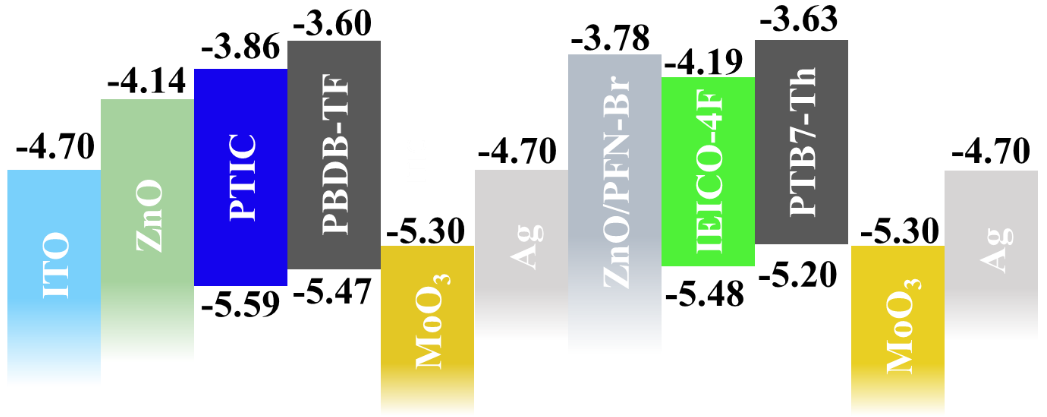

Supplementary Figure 31. Energy level diagram representing each layer in tandem OSCs.

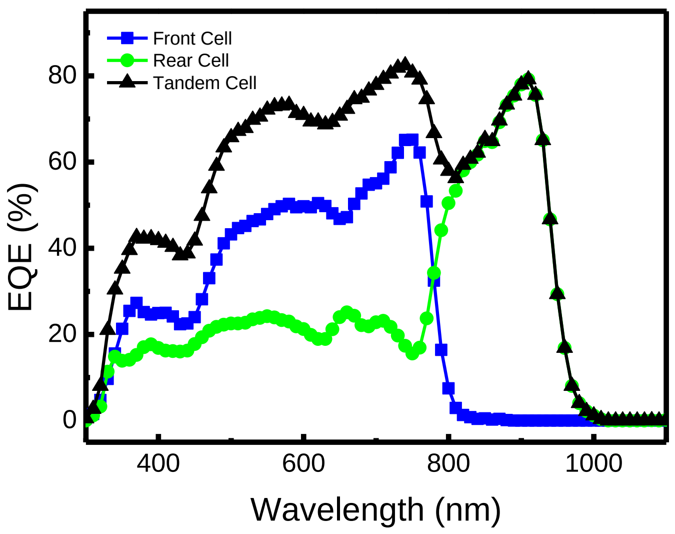

Supplementary Figure 32. The EQE for the tandem OSCs tested bias light.

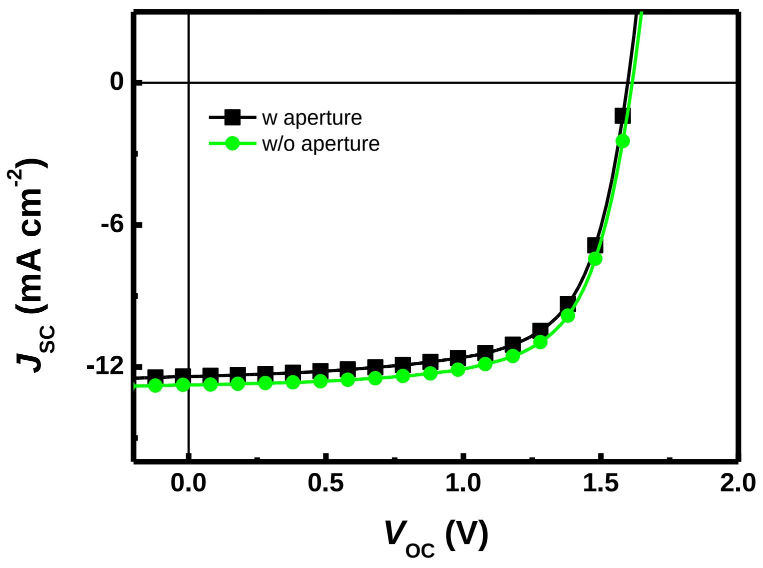

Supplementary Figure 33. The *J*-*V* curves for the tandem OSCs with and without aperture tested under AM 1.5G illumination (100 mW cm^{-2}).

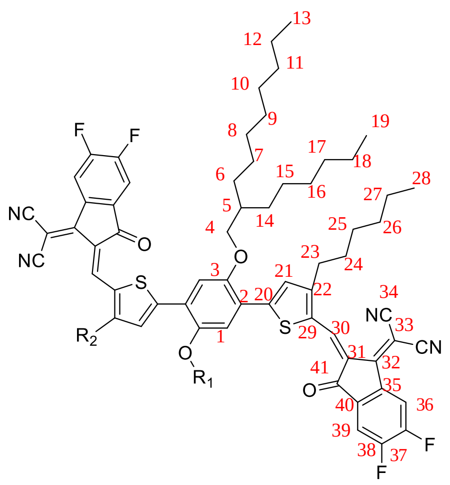

Supplementary Figure 34. The carbon-number of PTIC.

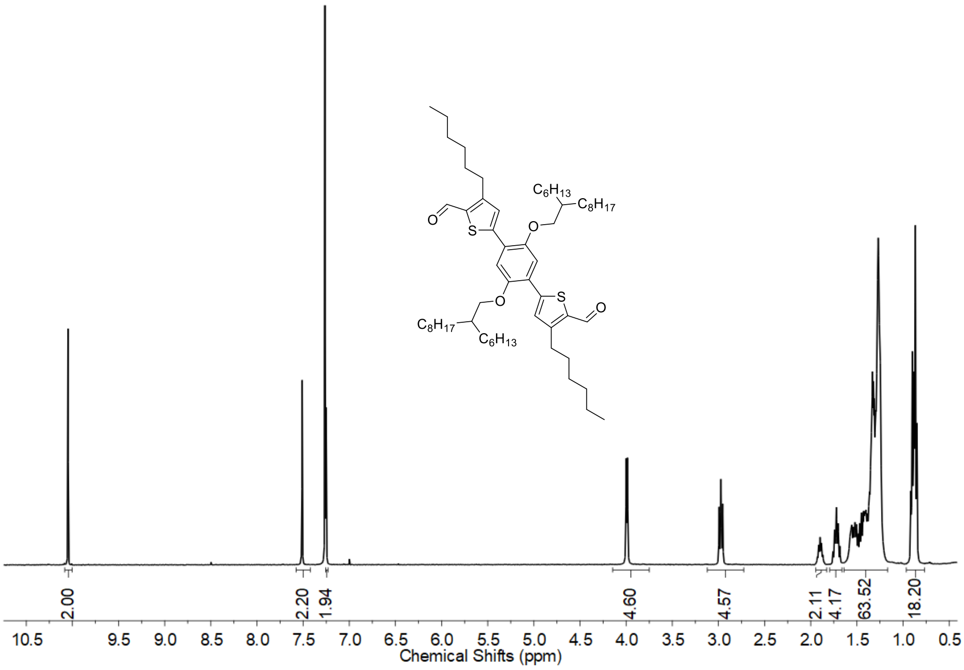

Supplementary Figure 35. The ^{1}H-NMR of PT-CHO in $CDCl_3$ at 25 °C.

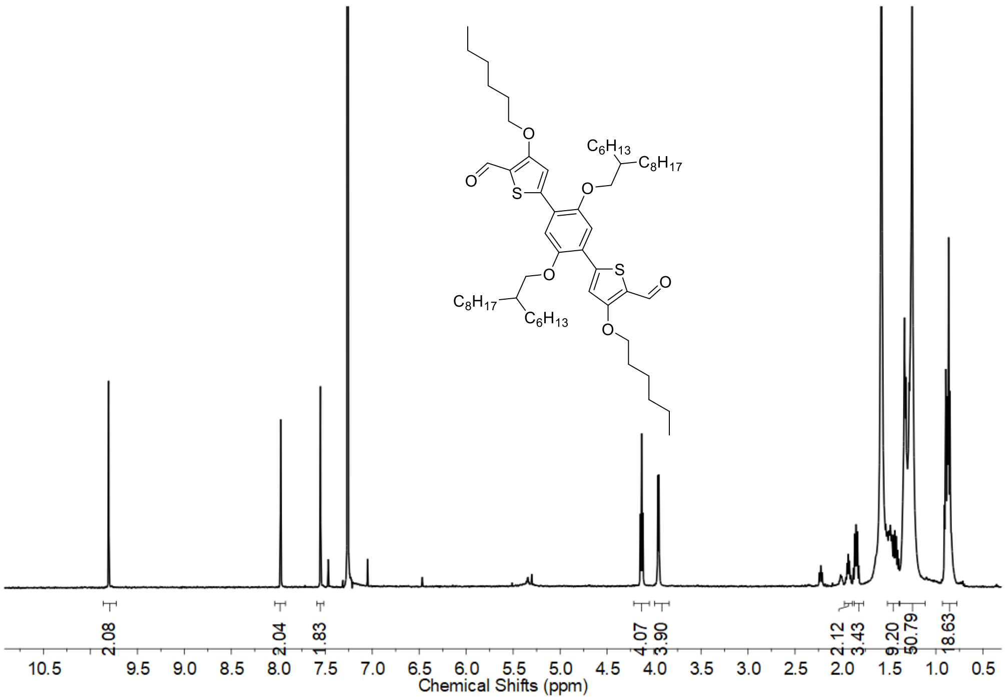

Supplementary Figure 36. The ^{1}H-NMR of PTO-CHO in $CDCl_3$ at 25 ℃.

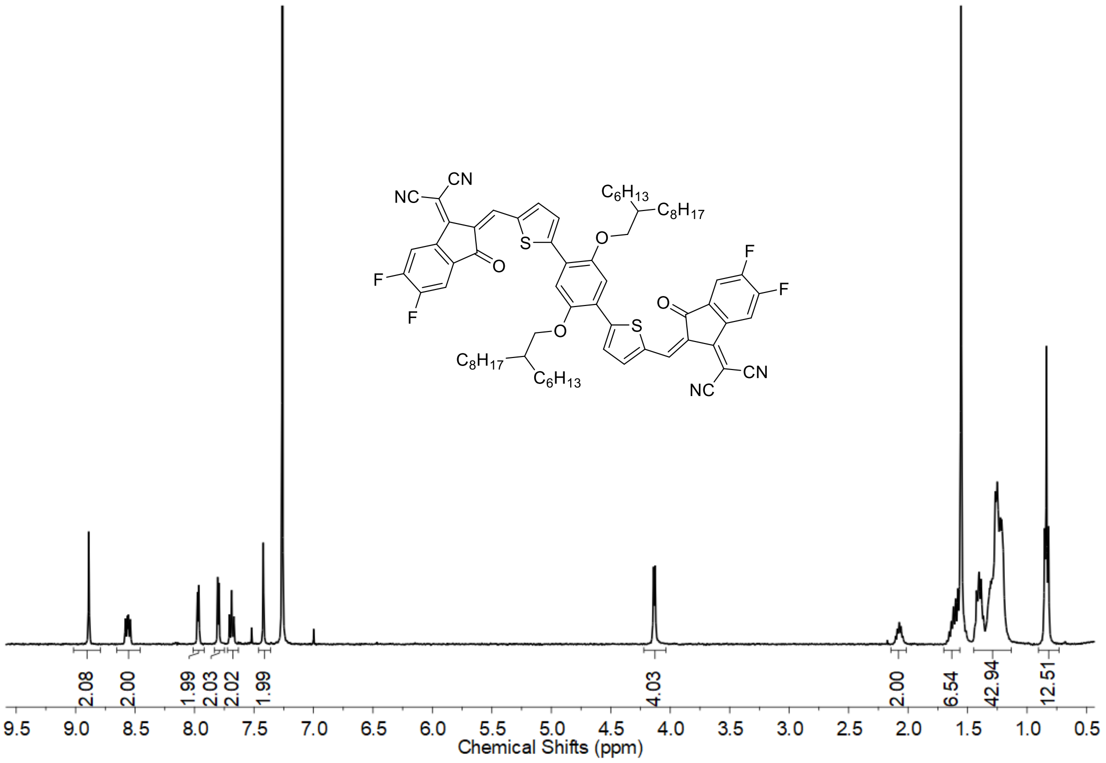

Supplementary Figure 37. The ^{1}H-NMR of PTICH in $CDCl_3$ at 25 ℃.

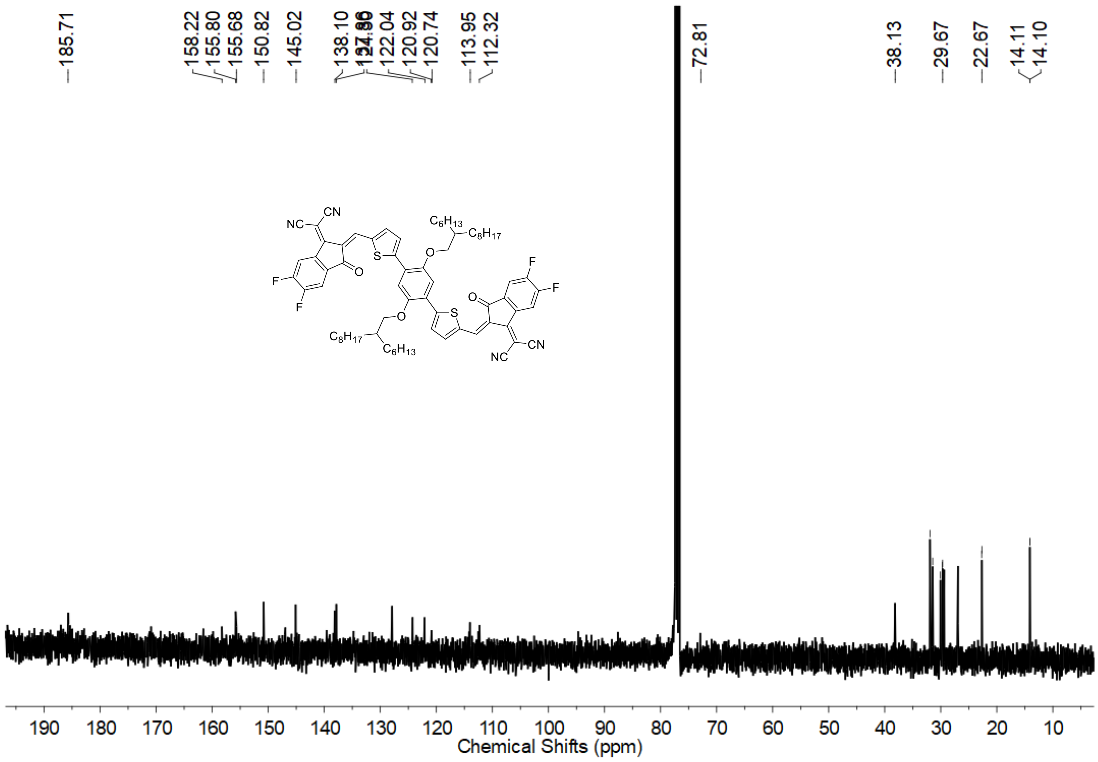

Supplementary Figure 38. The ^{13}C-NMR of PTICH in $CDCl_3$ at 25 ℃.

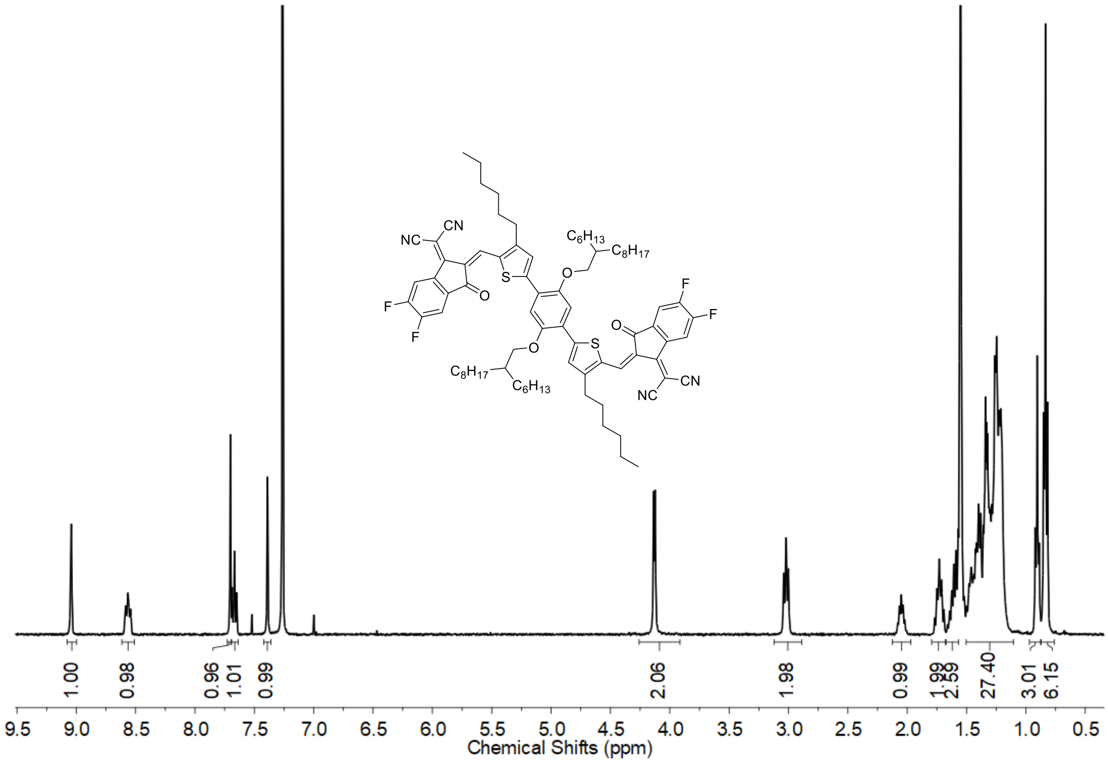

Supplementary Figure 39. The ^{1}H-NMR of PTIC in $CDCl_3$ at 25 ℃.

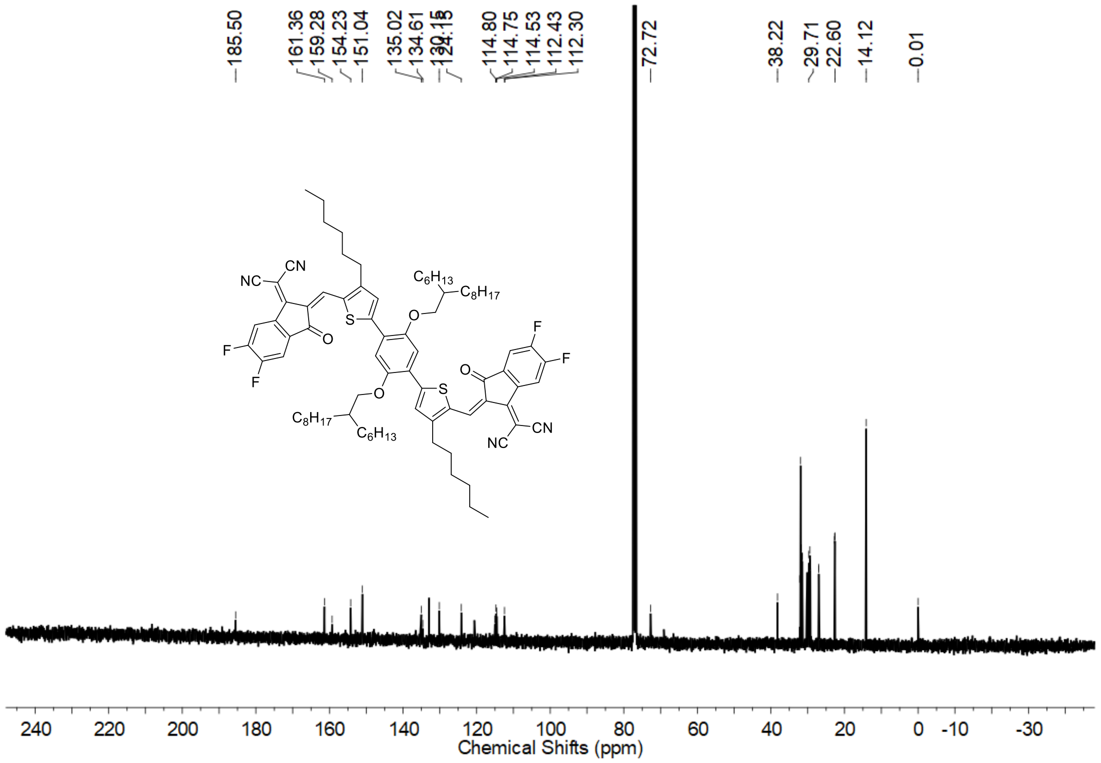

Supplementary Figure 40. The ^{13}C-NMR of PTIC in $CDCl_3$ at 25 ℃.

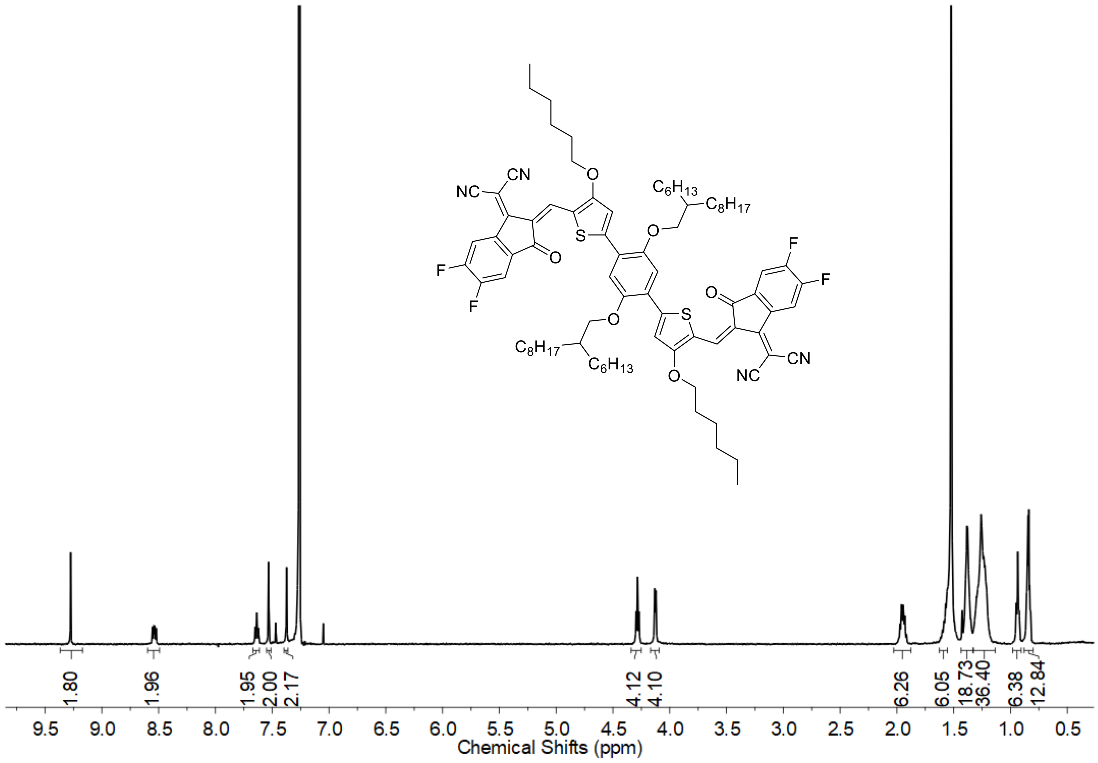

Supplementary Figure 41. The ^{1}H-NMR of PTICO in $CDCl_3$ at 25 ℃.

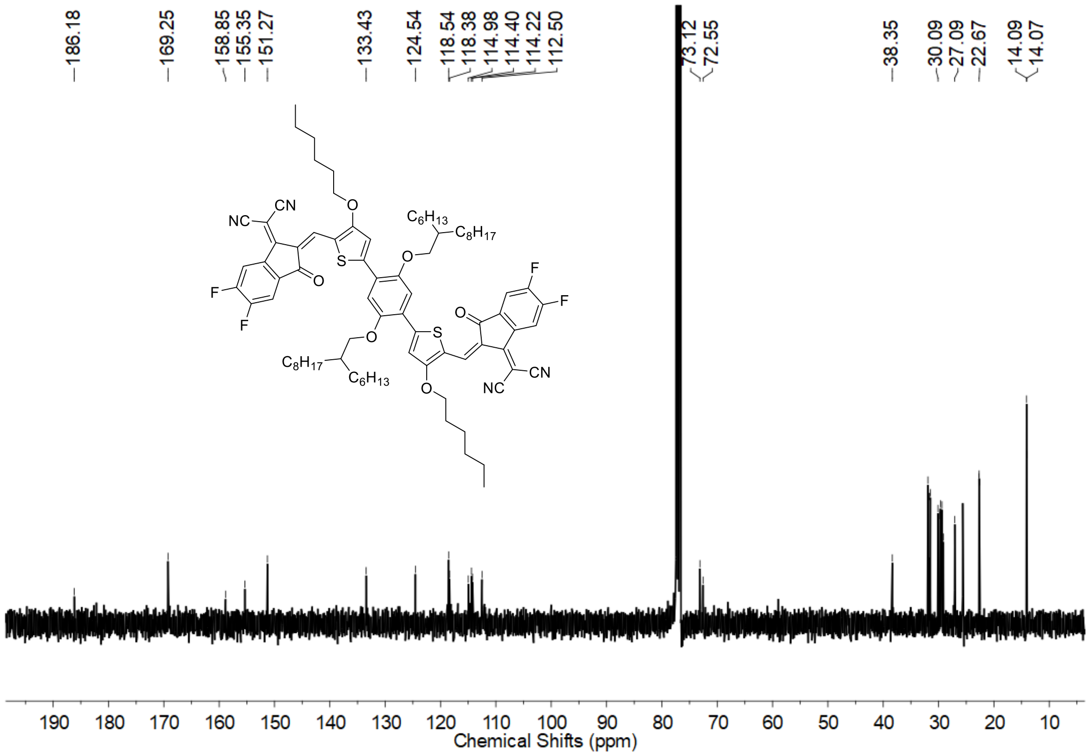

Supplementary Figure 42. The ^{13}C-NMR of PTICO in $CDCl_3$ at 25 ℃.

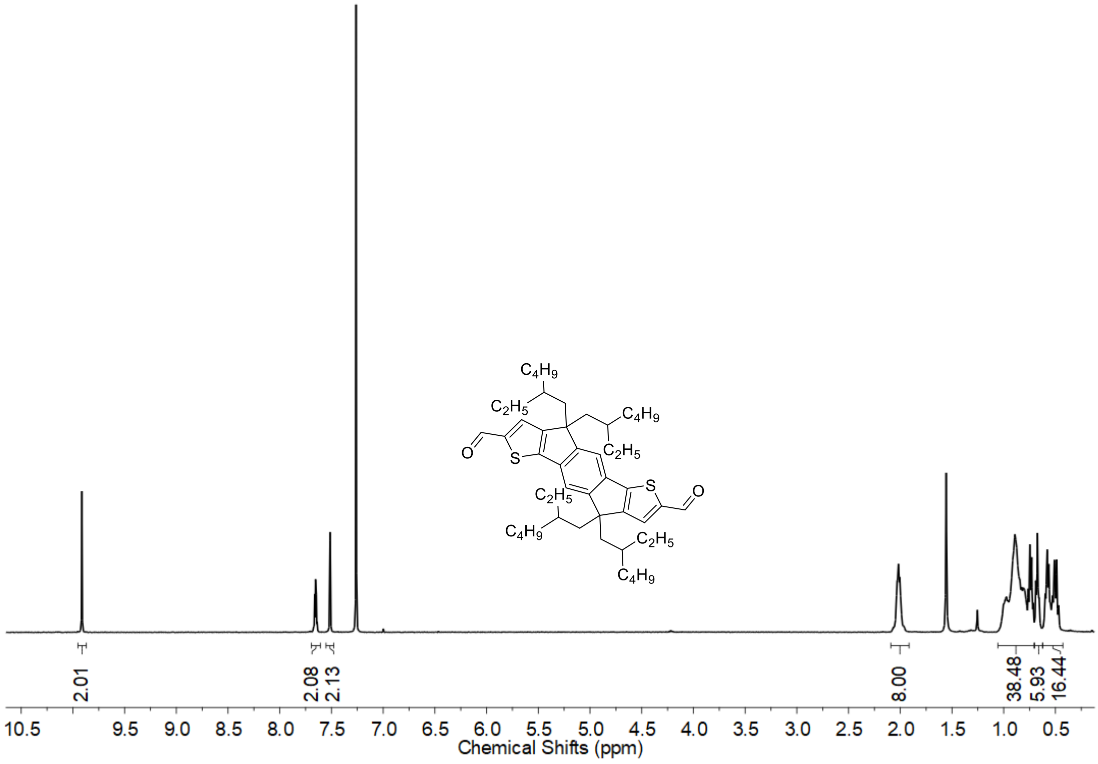

Supplementary Figure 43. The ^{1}H-NMR of IDTCHO in $CDCl_3$ at 25 ℃.

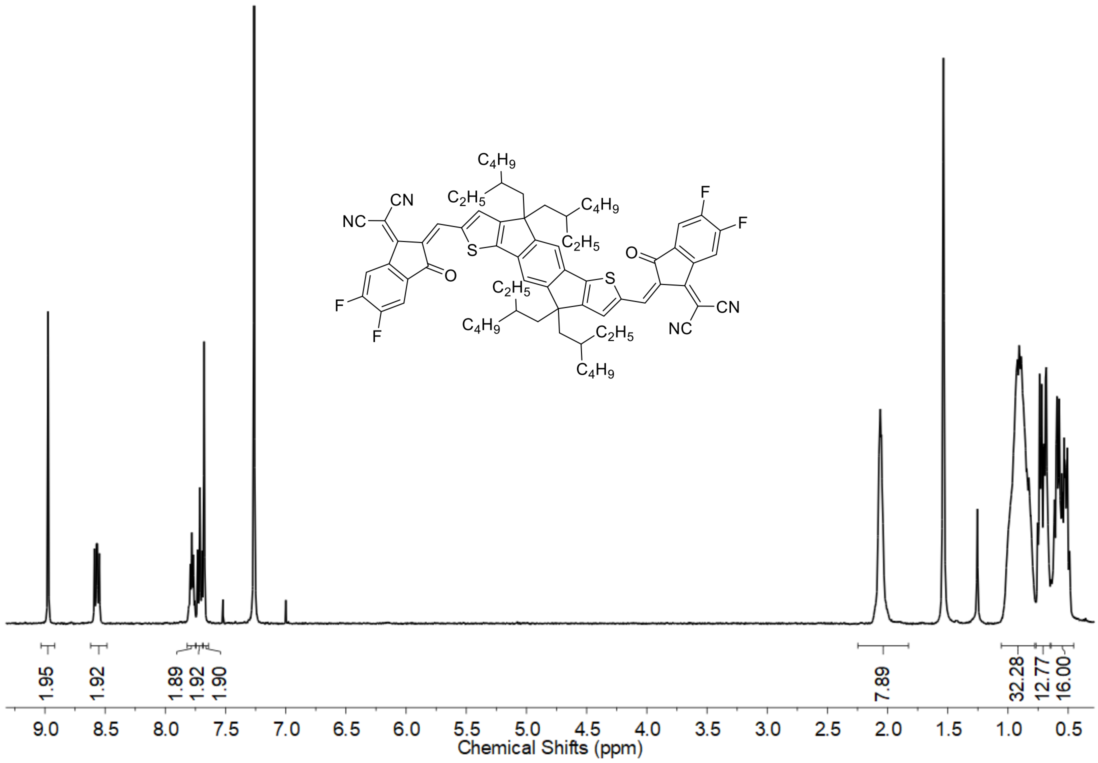

Supplementary Figure 44. The ^{1}H-NMR of ID4F in $CDCl_3$ at 25 ℃.

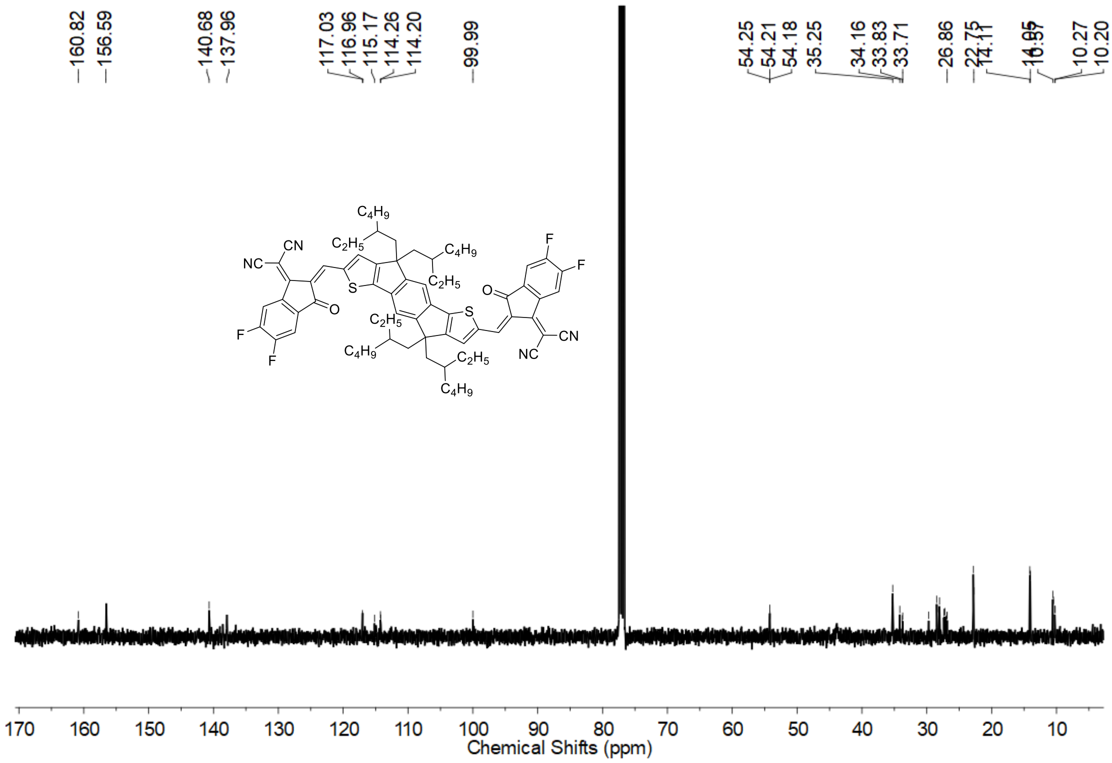

Supplementary Figure 45. The ^{13}C-NMR of ID4F in $CDCl_3$ at 25 ℃.

Supplementary Table 1. *SC* calculation for the investigated systems.[7]

		NSS	*RY*	*NCC*	*NUO*	*NHC*	*SC* index %
FERAs	***ITIC-2F***	*13*	*45.25*	*8*	*22*	*30*	*88.77*
	ITIC-M	*11*	*38.91*	*9*	*21*	*37*	*85.90*
	ITIC-DM	*11*	*41.32*	*9*	*21*	*37*	*80.46*
	ID4F	*15*	*48.31*	*9*	*24*	*32*	*97.15*
NFRAs	***PTIC***	*8*	*11.10*	*4*	*11*	*26*	*54.62*

[a] The synthetic complexity for the OSC materials, taking into account of five parameters including (1) the number of synthetic steps (*NSS*). Note that the synthesis DFIC terminal unit has been included into NSS for fair comparison with other FREAs; (2) The reciprocity yields (*RY*), (3) the number of unit operations (*NUO*) (in particular (4) the number of column chromatography (*NCC*)) required for the purification and (5) the number of hazardous chemicals used for their preparation (*NHC*). The *SC* is calculated according to the following Supplementary Equation 1:

$$SC = 35\frac{NSS}{NSS_{\max}} + 25\frac{\log(RY)}{\log(RY_{\max})} + 15\frac{NUO}{NUO_{\max}} + 15\frac{NCC}{NCC_{\max}} + 10\frac{NHC}{NHC_{\max}} \quad (1)$$

Where NSS_{max} =15; RY_{max} = 48.31; NUO_{max} = 24; NCC_{max} = 9; NHC_{max} = 37

Supplementary Table 2. The calculated F-C distance data according to REDOR spectra.

Chemical Shifts (ppm)	F-C Distance (nm)	Linked Proton	Possible assignment [a]
185.0	0.55	w/o	C_{41}
153.2	0.18	w/o	$C_{37/38}$
131.9	0.52	w	C_{30}
116.0	0.30-0.4	w	C_{21} or C_1
113.3	0.50	w	C_1 or C_{21}
110.0	0.26	w	$C_{36/39}$
74.4	0.43	w	C_4

[a] The carbon numbers were shown as **Supplementary Figure 34**.

Supplementary Table 3. The energy level calculated from CV and UPS, respectively.

	CV		UPS			
	HOMO[a] (eV)	LUMO[b] (eV)	V_b (eV)	SEC (eV)	HOMO[c] (eV)	LUMO[d] (eV)
PBDB-TF	-5.47	-3.60	1.43	36.22	-5.22	-3.42
ID4F	-5.83	-3.86	1.38	35.76	-5.62	-3.98
PTICH	-5.62	-3.80	1.57	36.12	-5.45	-3.85
PITC	-5.59	-3.81	1.58	36.16	-5.42	-3.89
PTICO	-5.48	-3.66	1.58	36.32	-5.25	-3.69

[a] The HOMO-Level according to CV was calculated as E_{HOMO} = -[E_{ox}-E(Fc/Fc$^+$)+4.8] eV. [b] The LUMO-Level according to CV was calculated as E_{LUMO} = -[E_{red}-E(Fc/Fc$^+$)+4.8] eV. [c] The HOMO-Level according to UPS was calculated as E_{HOMO} = 40-SEC+VB. [d] The LUMO-Level according to UPS was calculated as E_{LUMO} = E_{HOMO}+E_{opt}.

Supplementary Table 4. GIWAXS peaks for the pure and blend samples.

	Lamellar Peak /(*d* spacing) Å^{-1} / (Å)	π−π Peak /(*d* spacing) Å^{-1} /(Å)
ID4F	0.435 (14.4)	1.66 (3.79)^{c}
PTICH	0.355 (17.7)^{c}, 0.695$^{a, c}$, 1.05$^{b, c}$	1.73 (3.63)^{d}
PTIC	0.305 (20.6)^{c}, 0.600 $^{a, c}$, 0.885$^{b, c}$	1.75 (3.59)^{d}
PTICO	0.295 (21.3)^{c}, 0.575$^{a, c}$, 0.855$^{b, c}$	1.82 (3.45)^{d}
PBDB-TF: ID4F	0.305 (20.6), 0.420 (15.0)^{d}	1.66 (3.79)^{c}
PBDB-TF: PTICH	0.335 (18.8)^{c}, 0.680$^{a, c}$, 0.940$^{b, c}$	1.76 (3.57)^{d}
PBDB-TF: PTIC	0.305 (20.6)^{c}, 0.590$^{a, c}$, 0.885$^{b, c}$	1.76 (3.57)^{d}
PBDB-TF: PTICO	0.295 (21.3)^{c}, 0.570$^{a, c}$, 0.855^{b,c}	1.80 (3.49)^{d}
PBDB-TF	0.310 (20.3)	1.69 (3.72)

^{a}second order peaks, ^{b}third order peaks. ^{c}predominantly OOP features. ^{d}predominantly IP features.

Supplementary Methods

Materials All the chemicals including solvents, reagents, and catalysts were purchased from Sigma-Aldrich, Alfa Aesar chemical company, Sunatech Co., Ltd., and Derthon Optoelectronic Materials Science Technology Co LTD. Unless otherwise specified, such chemicals were used without any further purification.

General characterization. NMR spectra were recorded from Bruker Avance III (FT, DCH Cryoprobe, 400 MHz or 600 MHz for ^{1}H; 100 MHz or 126 MHz for ^{13}C) spectrometer under ambient temperature. UV-vis absorption spectra were recorded on a Shimadzu UV-2450 spectrophotometer. Cyclic voltammetry (CV) analysis were performed on a CHI600A electrochemical workstation with glass carbon electrode, Pt wire, and standard calomel electrode (SCE) as working electrode, counter electrode, and reference electrode, respectively, in a 0.1 M n-Bu_4NPF_6 in acetonitrile solution at the scan rate of 100 mV s^{-1}. The CV curves were recorded versus the potential of SCE, which was calibrated by the ferrocene-ferrocenium (Fc/Fc+) redox couple (5.1 eV below the vacuum level). The PL spectra measured using FluoroMax-4 HORIBA Jobin Yvon spectrofluorometer. Calculations were performed in the Density Functional Theory (DFT) framework as implemented in the GAUSSIAN software suite using the B3LYP exchange correlation functional and 6-31G(d) basis set.

SSNMR measurements. The experiments of SSNMR were performed on a Bruker Avance 400 III HD spectrometer (magnetic field strength 9.4 T) at resonance frequencies of 100.61 MHz for ^{13}C, 376.46 MHz for ^{19}F, and 400.13 MHz for ^{1}H. Magic angle spinning (MAS) experiments were performed on 3.2 mm MAS probes at

a spinning speed of 15 kHz. The ^{1}H and ^{13}C signals were referenced to those of adamantane at 1.8 ppm (^{1}H) and 38.5 ppm (^{13}C methylene), respectively. The ^{19}F signals was referenced to that of CF_3COONH_4 at 72.0 ppm. For cross-polarization magic angle spinning (CPMAS), the ^{13}C cross polarization contact time was 2.0 ms and the ^{1}H decoupling power was 104.02 kHz. For ^{1}H-^{13}C HETCOR (heteronuclear correlation), the cross polarization contact time was 180 us with the proton spin diffusion time of 10 μs and 10ms. For cross-polarization rotational-echo double resonance (CPREDOR), these experiments consisted of an initial $^{1}H\rightarrow^{13}C$ cross-polarization (CP) followed by the $^{19}F/^{13}C$ rotational-echo double resonance (REDOR) experiment during which ^{1}H decoupling was applied. At each increment of NTr (rotor cycle), two spectra were collected: a reference spectrum in which the dephasing π pulses on ^{19}F are not applied (S_0) and a dephased spectrum in which these dephasing pulses are applied (S). Each REDOR curve consists of a plot of S/S_0 containing 11 points. The $^{1}H\rightarrow^{13}C$ CP contact time was 4 ms, the ^{13}C π refocusing pulses were 6.2 μs, and the ^{19}F π dephasing pulses were 4.4 μs. Five hundred to a thousand scans were collected for these spectrums.

PL Quantum Efficiencies. The absolute PL quantum efficiencies of the samples were measured with a system consisting of a Xenon lamp, optical fiber, a QE65000 spectrometer (Ocean Optics) and a home-designed integrating sphere. The PL quantum efficiencies of ID4F, PTICH, PTIC and PTICO were 1.96%, 1.07%, 1.23% and 1.82% in chlorobenzene solution, and were 3.73%, 4.63%, 7.11% and 5.69% in films, respectively.

SCLC measurement. The charge carrier mobilities were measured using the space-charge-limited current (SCLC) method. Hole-only devices were fabricated in a structure of ITO/PEDOT:PSS/Active Layer/MoO3/Ag, electron-only devices were fabricated in a structure of ITO/ZnO/Active Layer/PFN-Br/Ag. The device characteristics were extracted by modeling the dark current under forward bias using the SCLC expression described by the Mott-Gurney law (**Supplementary Equation 2**):

$$J = \frac{9}{8}\varepsilon_r\varepsilon_0\mu\frac{V^2}{L^3} \qquad (2)$$

Here, $\varepsilon_r \approx 3$ is the average dielectric constant of the blend film, ε_0 is the permittivity of the free space, μ is the carrier mobility, L ≈ 100 nm is the thickness of the film, and V is the applied voltage.

AFM characterization. The samples were fabricated same with OSC conditions. Topographic and phase images of films were obtained on a VeecoMultiMode AFM in the tapping mode, and the scanning rate for a 2.5 μm × 2.5 μm image size was 1.5 Hz.

GIWAXS measurements. GIWAXS were carried out with a Xeuss 2.0 SAXS/WAXS laboratory beamline using a Cu X-ray source (8.05 keV, 1.54 Å) and Pilatus3R 300K detector. The incidence angle is 0.2 °.

Preparation of NFRAs and ID4F.

The intermediates 1,4-dibromo-2,5-bis((2-hexyldecyl)oxy)benzene[1], 3-hexyl-2-formyl-thiophene[2] and 3-(hexyloxy)-2-formylthiophene[3] were synthesized as reported.

General Synthesis of Non-Fused PT Core

In the Ar atmosphere, 1,4-dibromo-2,5-bis((2-hexyldecyl)oxy)benzene (0.20 mmol), 3-substitutional-2-formylthiophene (0.60 mmol) and trimethylacetic acid (0.05 mmol), $PCy_3 \cdot HBF_4$ (2 mol%) were dissolved into dry toluene (10 mL). $Pd(PPh_3)_4$ (1 mol%) and K_2CO_3 (0.3 mmol) were added into reaction mixture and then the solution was heating at 80 ℃ for 16 hours. Then the solvent was removed in vacuo. The residue was further purified by using column chromatography to obtain the desired non-fused PT backbone.

PTH-CHO The synthesis is following the general protocol with yield of 70%.[1]

PT-CHO The synthesis is following the general protocol with yield of 61%. ^{1}H-NMR ($CDCl_3$, 400 MHz, 25 °C): 10.05 (s, 2H), 7.51 (s, 2H), 7.25 (s, 2H), 4.00–3.99 (d, 4H, *J* = 5.2 Hz), 2.99–2.95 (t, 4H, *J* = 7.8 Hz), 1.93–1.87 (m, 4H), 1.76–1.68 (m, 8H), 1.57–1.29 (m, 64H), 0.92–0.85 (m, 18H).

PTO-CHO The synthesis is following the general protocol with yield of 31%. ^{1}H-NMR ($CDCl_3$, 400 MHz, 25 °C): 9.80 (s, 2H), 7.97 (s, 2H), 7.55 (s, 2H), 4.15–4.12 (t, 4H, *J* = 5.2 Hz), 3.96–3.95 (d, 4H, *J* = 4.4 Hz), 1.95–1.91 (m, 2H), 1.87–1.82 (m, 9H), 1.48–1.25 (m, 50H), 0.91–0.85 (m, 18H)

General Synthesis of NFRAs

The non-fused core, 5,5'-(2,5-bis((2-hexyldecyl)oxy)-1,4-phenylene) bis(3-substituent-2-thenaldehyde) (*i.e.* PTH-CHO or PT-CHO or PTO-CHO) (0.20 mmol), fluorinated 1,1-dicyanomethylene-3-indanone (DFIC, 0.50 mmol) and a few drops of pyridine was dissolved in to chloroform ($CHCl_3$). The reaction mixture was refluxed overnight, and then concentrated in vacuo. The solid crude products were

obtained through methanol precipitation, and washed with methanol and acetone, respectively. The final crystalline solid was obtained through the re-crystallization from $CHCl_3$ and methanol, and washed with the mixture of $CHCl_3$/methanol (v/v = 1/1) to obtain pure product.

PTICH The synthesis is following the general protocol with yield of 78%. ^{1}H-NMR ($CDCl_3$, 400 MHz, 25 ℃): 8.89 (s, 2H), 8.58–8.54 (m, 2H), 7.97–7.96 (d, 2H, *J* = 4.8 Hz), 7.80–7.79 (d, 2H, *J* = 4.8 Hz), 7.71–7.66 (m, 2H), 7.42 (s, 2H), 4.14–4.13 (d, 4H, *J* = 5.6 Hz), 2.11–2.04 (m, 2H), 1.67–1.58 (m, 8H), 1.42–1.38 (m, 8H), 1.29–1.21(m, 32H), 0.85–0.82 (m, 12H). ^{13}C-NMR ($CDCl_3$, 126 MHz, 25 ℃): δ (ppm): 185.71, 158.22, 155.80, 155.68, 150.82, 146.86, 145.02, 139.47, 138.10, 137.86, 127.94, 124.30, 122.04, 120.92, 120.74, 113.95, 112.32, 72.81, 38.13, 31.93, 31.91, 31.43, 30.06, 29.72, 29.67, 22.70, 22.67, 14.11, 14.10. MS (MALDI-TOF): Calcd for $C_{72}H_{79}F_4N_4O_4S_2$ $[M+H]^+$, 1204.55; found: 1204.71.

PTIC The synthesis is following the general protocol with yield of 70%. ^{1}H-NMR ($CDCl_3$, 400 MHz, 25 °C): 9.01 (s, 2H), 8.51-8.55 (m, 2H), 7.69 (s, 2H), 7.67-7.64 (m, 2H), 7.37 (s, 2H), 4.14–4.13 (d, 4H, *J* = 5.2 Hz), 3.03–2.99 (t, 4H, *J* = 7.8 Hz), 2.09–2.03 (m, 2H), 1.76–1.69 (m, 4H), 1.65–1.60 (m, 6H), 1.47–1.21 (m, 54H), 0.92–0.89 (m, 6H), 0.85–0.82 (m, 12H). ^{13}C-NMR ($CDCl_3$, 100 MHz, 25 °C): δ (ppm): 185.50, 161.41, 159.25, 154.23, 151.04, 134.99, 134.59, 132.93, 130.09, 124.21, 120.64, 115.01, 114.80, 114.75, 114.58, 112.48, 112.43, 112.27, 72.65, 38.22, 32.12, 31.94, 31.70, 31.44, 30.30, 30.13, 29.71, 29.42, 29.29, 27.02, 27.00, 22.72, 22.68, 22.60, 14.12. MS (MALDI-TOF): Calcd for $C_{84}H_{103}F_4N_4O_4S_2$ $[M+H]^+$, 1371.73;

found: 1371.92.

PTICO The synthesis is following the general protocol with yield of 62%. ^{1}H-NMR ($CDCl_3$, 400 MHz, 25 °C): 9.28 (s, 2H), 8.56–8.52 (m, 2H), 7.65–7.62 (m, 2H), 7.53 (s, 2H), 7.37 (s, 2H), 4.30–4.27 (t, 4H, *J* = 5.2 Hz), 4.13–4.12 (t, 4H, *J* = 3.6 Hz), 1.97–1.92 (m, 6H), 1.61–1.54 (m, 6H), 1.39–1.37 (m, 18H), 1.30–1.21 (m, 36H), 0.95–0.92 (m, 6H), 0.86–0.84 (m, 12H). ^{13}C-NMR ($CDCl_3$, 100 MHz, 25 °C): δ (ppm): 186.18, 169.25, 158.92, 158.85, 155.35, 151.35, 151.27, 133.43, 124.54, 118.54, 118.38, 114.98, 114.88, 114.40, 114.22, 112.50, 73.12, 72.55, 38.35, 31.92, 31.89, 31.65, 31.49, 30.09, 29.74, 29.66, 29.38, 29.14, 27.09, 27.06, 25.63, 22.69, 22.67, 22.59, 14.09, 14.07. MS (MALDI-TOF): Calcd for $C_{84}H_{102}F_4N_4O_6S_2$ $[M+H]^+$, 1403.73; found: 1403.87.

Synthesized Route of ID4F

Synthesized of **IDT-CHO**

The $POCl_3$ was added to dry DMF dropwise at 0 °C. After an hour, the $C_2H_4Cl_2$ solution of intermediate **IDT**[4] (200 mg, 0.28 mmol) was added one-pot and the mixture was heating at 80 ℃ overnight. The solution was then pull into amount of water. The isolated organic layer was washed with water, Na_2CO_3 aqueous solution and brine, subsequently. The organic phase was dried over Na_2SO_4, filtrated, and evaporated to dryness under reduced pressure. The crude product was further purified by column chromatography (SiO_2, petroleum ether/ dichloromethane = 1/2) to afford

IDT-CHO as a yellow powder (148 mg, 72% yield) ^{1}H-NMR ($CDCl_3$, 400 MHz, 25 ℃): 9.91 (s, 2H), 7.67–7.64 (m, 2H), 7.51 (s, 2H), 2.02–2.00 (m, 2H), 1.00–0.78 (m, 38H), 0.70–0.60 (m, 6H), 0.60–0.47 (m, 16H).

Synthesis of **ID4F**

The **IDT-CHO** (154 mg, 0.20 mmol), DFIC (138 mg, 0.60 mmol) and few drops of pyridine was dissolved in to $CHCl_3$. The mixture was then reflux overnight. The solution was concentrated in vacuo. The crude products were obtained through methanol precipitation, and then filtration. The solid was washed with methanol and acetone. and then dissolved into $CHCl_3$ and re-crystalized by $CHCl_3$ and methanol. The crystalline solid was washed with the mixture of $CHCl_3$/methanol (v/v = 1/1) to obtain pure product. ^{1}H-NMR ($CDCl_3$, 400 MHz, 25 ℃): 8.97 (s, 2H), 8.59–8.55 (m, 2H), 7.79–7.77 (m, 2H), 7.73-7.69 (m, 2H), 7.68 (m, 2H), 2.09–2.01 (m, 8H), 1.00–0.81 (m, 34H), 0.75–0.68 (m, 14H), 0.61–0.49 (m, 16H). ^{13}C-NMR ($CDCl_3$, 400 MHz, 25 ℃): 160.92, 156.59, 140.68, 137.96, 117.03, 116.69, 115.17, 114.26, 114.20, 99.99, 54.25, 54.21, 54.18, 35.31, 35.27, 35.25, 35.21, 34.16, 33.83, 33.71, 29.71, 28.38, 28.04, 27.19, 27.04, 26.88, 26.86, 22.83, 22.78, 22.75, 14.11, 14.05, 14.05, 10.57, 10.46, 10.27, 10.20. MS (MALDI-TOF): Calcd for $C_{78}H_{79}F_4N_4O_2S_2$ $[M+H]^+$, 1195.56; found: 1195.87.

Supplementary References

1. Zhang Zhongqiang*, et al.* A Simple Electron Acceptor with Unfused Backbone for Polymer Solar Cells. *Acta Phys -Chim Sin* **35**, 7 (2019).
2. Kang S-H, Lee HR, Dutta GK, Lee J, Oh JH, Yang C. A Role of Side-Chain Regiochemistry of Thienylene–Vinylene–Thienylene (TVT) in the Transistor Performance of Isomeric Polymers. *Macromolecules* **50**, 884-890 (2017).
3. Yang Y*, et al.* Novel chromophores with excellent electro-optic activity based on double-donor chromophores by optimizing thiophene bridges. *Dyes and Pigments* **122**, 139-146 (2015).
4. Bronstein H*, et al.* Indacenodithiophene-co-benzothiadiazole Copolymers for High Performance Solar Cells or Transistors via Alkyl Chain Optimization. *Macromolecules* **44**, 6649-6652 (2011).
5. Yao Z*, et al.* Dithienopicenocarbazole-Based Acceptors for Efficient Organic Solar Cells with Optoelectronic Response Over 1000 nm and an Extremely Low Energy Loss. *J Am Chem Soc* **140**, 2054-2057 (2018).
6. Shi X*, et al.* Design of a Highly Crystalline Low-Band Gap Fused-Ring Electron Acceptor for High-Efficiency Solar Cells with Low Energy Loss. *Chem Mater* **29**, 8369-8376 (2017).
7. Po R, Bianchi G, Carbonera C, Pellegrino A. "All That Glisters Is Not Gold": An Analysis of the Synthetic Complexity of Efficient Polymer Donors for Polymer Solar Cells. *Macromolecules* **48**, 453-461 (2015).
