# Supplementary material for: Simple non-fused electron acceptors for efficient and stable organic solar cells
Source: Nat Commun. 2019 May 14;10:2152. doi: 10.1038/s41467-019-10098-z (PMC6517432; doi:10.1038/s41467-019-10098-z)
Supplement: Supplementary file 2 — Solar Cells Reporting Summary [file 41467_2019_10098_MOESM2_ESM.pdf]

Corresponding author(s): *Double-blind peer review submissions: write DBPR and your manuscript number here instead of author names.*

Solar Cells Reporting Summary

Nature Research wishes to improve the reproducibility of the work that we publish. This form is intended for publication with all accepted papers reporting the characterization of photovoltaic devices and provides structure for consistency and transparency in reporting. Some list items might not apply to an individual manuscript, but all fields must be completed for clarity.

For further information on Nature Research policies, including our data availability policy, see Authors & Referees.

Experimental design

Please check: are the following details reported in the manuscript?

1. Dimensions

Item	Yes	No	Details
Area of the tested solar cells	☒	☐	Method section in MS
Method used to determine the device area	☒	☐	Method section in MS

2. Current-voltage characterization

Item	Yes	No	Details
Current density-voltage (J-V) plots in both forward and backward direction	☒	☐	Figures in MS and SI
Voltage scan conditions *For instance: scan direction, speed, dwell times*	☒	☐	Method section in MS
Test environment *For instance: characterization temperature, in air or in glove box*	☒	☐	Method section in MS
Protocol for preconditioning of the device before its characterization	☒	☐	Method section in MS
Stability of the J-V characteristic *Verified with time evolution of the maximum power point or with the photocurrent at maximum power point; see ref. 7 for details.*	☒	☐	Method section in MS

3. Hysteresis or any other unusual behaviour

Item	Yes	No	Details
Description of the unusual behaviour observed during the characterization	☐	☒	polymer solar cells are known without hysteresis behavior
Related experimental data	☐	☒	polymer solar cells are known without hysteresis behavior

4. Efficiency

Item	Yes	No	Details
External quantum efficiency (EQE) or incident photons to current efficiency (IPCE)	☒	☐	Figure and method section in MS
A comparison between the integrated response under the standard reference spectrum and the response measure under the simulator	☒	☐	Figure and discussion in MS
For tandem solar cells, the bias illumination and bias voltage used for each subcell	☒	☐	Method section in MS

5. Calibration

Item	Yes	No	Details
Light source and reference cell or sensor used for the characterization	☒	☐	Method section in MS
Confirmation that the reference cell was calibrated and certified	☒	☐	Method section in MS

Calculation of spectral mismatch between the reference cell and the devices under test

- [x] Yes
- [ ] No

Method section in MS

6. Mask/aperture

Size of the mask/aperture used during testing

- [x] Yes
- [ ] No

Method section in MS

Variation of the measured short-circuit current density with the mask/aperture area

- [x] Yes
- [ ] No

Method section in MS and SI

7. Performance certification

Identity of the independent certification laboratory that confirmed the photovoltaic performance

- [ ] Yes
- [x] No

Have been tested under standard condition and setups with certified parts, therefore without the necessary of third-party certification.

A copy of any certificate(s)
Provide in Supplementary Information

- [ ] Yes
- [x] No

Have been tested under standard condition and setups with certified parts, therefore without the necessary of third-party certification.

8. Statistics

Number of solar cells tested

- [x] Yes
- [ ] No

figure and table in MS

Statistical analysis of the device performance

- [x] Yes
- [ ] No

figure and table in MS

9. Long-term stability analysis

Type of analysis, bias conditions and environmental conditions
For instance: illumination type, temperature, atmosphere humidity, encapsulation method, preconditioning temperature

- [x] Yes
- [ ] No

figure and table in MS
